# Development and validation of random-forest based federated ensemble learning algorithms for delirium prediction using electronic medical records from eleven hospitals in Austria: a retrospective study

**DOI:** 10.1186/s12911-025-03322-y

**Published:** 2026-01-14

**Authors:** Sai Pavan Kumar Veeranki, Dieter Hayn, Diether Kramer, Piyush Gajananrao Gampawar, Martin Baumgartner, Lena Delia Lorenzer, Michael Schrempf, Günter Schreier

**Affiliations:** 1 Institute of Neural Engineering, Graz University of Technology, Stremayrgasse 16/IV, Graz, 8010 Austria; 2 Steiermärkische Krankenanstaltengesellschaft M.B.H. (KAGes), Billrothgasse 18a, Graz, 8010 Austria; 3 AIT Austrian Institute of Technology, Center for Health & Bioresources, Reininghausstraße 13, Graz, 8020 Austria; 4 Division of Cardiology, Medical University of Graz, Auenbruggerplatz 15, Graz, 8036 Austria; 5 Research Unit-Genetic Epidemiology, Gottfried Schatz Research Centre for Cell Signaling, Metabolism and Aging, Molecular Biology and Biochemistry, Medical University of Graz, Auenbruggerplatz 2, Graz, 8036 Austria; 6 Ludwig Boltzmann Institute for Digital Health and Prevention, Lindhofstrasse 22, Salzburg, 5020 Austria; 7 PH Predicting Health GmbH, Alberstrasse 17, Graz, 8010 Austria

**Keywords:** Federated learning, Ensemble learning, Delirium prediction, Electronic medical records, Random forest

## Abstract

**Background:**

Machine learning models have shown great potential in preventive medicine but require large datasets, which is a challenge due to strict privacy regulations in the healthcare sector. Federated learning is an approach that enables collaboration between institutions while preserving data privacy. The focus today in research is highly on developing federated learning methods using artificial neural networks. In this study, we aimed to contribute federated learning modelling methods applied for random forests with an use-case of predicting delirium in hospitalised patients using data from multiple hospitals.

**Methods:**

We collected data from eleven hospitals, including 29,479 patients and 627 features. We developed individual random forest models for each hospital data and a general model using all data. We developed federated learning models by averaging the predictions of the individual hospital models, with different schemes based on the number of samples, positives cases, minority cases and maximum possible diversity and evaluated the models using area under the receiver operating characteristic curve (AUROC).

**Results:**

The general model outperformed all the other models with an AUROC of 0.855 [0.845–0.865]. Models trained on data from single hospitals varied in performance with an AUROC ranging from 0.633 to 0.829. Models from hospitals with large datasets performed better than those of small hospitals. Federated learning models outperformed individual models. With an AUROC of 0.794 [0.782–0.806], unweighted averaging achieved the worst results. Among the weighting algorithms, the number of positive cases performed the best reaching an AUROC of 0.843 [0.832–0.854], followed by minority cases (AUROC = 0.841 [0.830–0.852]), maximum possible diversity (AUROC = 0.836 [0.825–0.847]) and number of samples (AUROC = 0.830 [0.819–0.841]).

**Conclusions:**

Results show that federated learning models can perform better than hospital-specific models in some cases, especially hospitals with limited data. In case of datasets of different size, we suggest weighted averaging based on the number of samples. If the datasets are class imbalanced, minority cases or maximum possible diversity should also be considered. Additionally, federated learning models maintain consistency compared to hospital specific models.

**Clinical trial registration:**

Not applicable.

**Supplementary information:**

The online version contains supplementary material available at 10.1186/s12911-025-03322-y.

## Background

Application of artificial intelligence (AI), particularly machine learning (ML), has led to considerable achievements in healthcare [1]. In traditional ML approaches, the contributing research groups share their data on a centralized server, which is associated with certain privacy and data protection issues when health data are concerned [2]. Therefore, anonymisation and pseudonymisation are commonly used, whereas K-anonymity and differential privacy are popular approaches to quantify the privacy of data [3, 4]. Even when the data are anonymised and shared in a centralised server, data linkage is a tedious task [5]. Centralised data servers may also lead to insecure storage [6–10].

Federated Learning (FL) is a technique in which ML algorithms train at multiple collaborators on local data without sharing the data and share the trained models instead. In the article ‘The future of digital health with federated learning’ authors explained, two FL workflows, centralised and peer-to-peer aggregations. In the former, models are trained on local data and are collected at an aggregation server and the final model is distributed with the partners, where as in the latter, the local models are distributed with partners and aggregated locally. Possible topologies and computing plans were also discussed [11]. The main idea is to train ML models on the datasets that are distributed across multiple devices/institutions while preserving the data leakage. Thus, FL is defined as a learning system that is a learning process in which the data owners collaboratively train a model in which data owners do not expose their data to others [12]. FL further has been categorised based on the feature and sample space. Horizontal FL, where the feature space remains the same, samples characteristics differ, whereas in vertical FL, sample characteristics remain the same but feature space changes. Federated transfer is another category where the datasets differ in both sample characteristics and features.

### Related work

Google coined the term FL in their blog post in 2014 discussing about learning the keyboard using patterns of mobile users. The idea was to train ML models based on the datasets distributed across multiple devices that preserve the users’ privacy by preventing the data transfer. Ever since, there is a surge in developing various FL methods for linear, non-linear and tree based approaches. In this chapter, we tried to summarise state of the art of the FL methods dealing with real time challenges.

Initial FL research and many ongoing efforts have predominantly focused on developments of Artificial Neural Networks (ANN) such as FedAvg [13] for model aggregation, and FedPAQ [14] and FedSVRG [15] for enhancing communication efficiency, robustness and advanced privacy among many others. These approaches leverage on stochastic gradient decent for optimising the loss functions being FedAvg as a popular FL method. Variations of updating the weights were discussed to improve the performance of the models in FedRobust [16], FedMA [17]. Many of these techniques can be applied to differential models [18]. The application of FL to increase the privacy, a layer of privacy mechanism such as differential privacy, hashing and cryptographic methods [6] over the models applied while distribution [19–25].

In one of our recent publications, we analysed multiple FL schemes to predict cardiac malfunctions based on ANN using electrocardiograms as dataset [26]. Khan et al. introduced an asynchronous FL approach using Deep Neural Networks (DNNs) for cardiovascular disease prediction, focusing on asynchronous updates and temporally weighted aggregation of DNN parameters to improve communication cost and model accuracy [27].

Beyond deep learning, federated approaches are being applied with various ML models for clinical prediction tasks. For instance, Sujit et al. demonstrated a FL framework for heart disease prediction using a l1-regularised Support Vector Machine (SVM), achieving high accuracy showing the general interest in FL for cardiovascular risk assessment [28].

However, while deep learning FL is advancing rapidly, Decision Tree (DT) based methods such as random forest (RF), Gradient boosting Decision Trees (GBDT) are efficient in inference, computational efficiency and interpretability particularly for structured Electronic Medical Record (EMR) data. In our previous analyses, tree-based-models in general and RF models in particular outperformed other ML methods, including ANNs while using EMRs to predict delirium [29–31]. RFs are a proven consistent ML method, especially for structured healthcare data such as EMRs [29–31], which require little computational resources and are transparent to explain [32].

Existing works on tree-based FL includes frameworks for vertical FL settings like Federated Forest by Yang Liu et al., which used publicly available target marketing data to train models at individual institutions in parallel and updated the split criteria at each node for each tree to build a forest in a federated setting [33]. The same authors have proposed a federated extreme boosting learning framework for mobile crowd-sensing [34]. SecureBoost is another framework proposed by Cheng et al. in the vertical FL setting using GBDTs [35]. They have applied homomorphic encryption to pretext the gradients. Similar work was published by Markovic et al. on intrusion detection [36]. Another algorithm called boosting based federated random forest (BOFRF) is an interesting concept of assigning weights to the DTs by calculating confusion matrices using test data from all the sites involved [37]. There are methods that discards few DTs randomly before combining into a single RF or GBDT. Hauschild et al. used cancer data from five datasets and trained local RF models, which were then combined to a single RF model [38].

Recent advancements continue to validate and expand the use of tree-based ensembles in horizontal FL for various clinical prediction tasks. Henshaw et al. developed an FL based asthma prediction model for children using XGBoost, another tree based ensemble. Their approach, implemented with the FATE framework [39], employed federated averaging algorithm and demonstrated performance comparable to existing centralised ML models for asthma prediction [40]. Further, Gifsy et al. proposed a federated forest approach for diabetes mellitus prediction. They combined locally trained RF models in a horizontal FL setting, utilising SMOTE [41] for class imbalance and CGANS [42] for non-iid data simulation, reporting exceptionally high performance on a public diabetes dataset sourced from mendeley data [43]. Meduri et al. presented an FL framework for rare diseases where RFs achieved the best performance among several ML models, effectively handling complex and imbalanced multi-institutional EMR data [44].

The main challenge in horizontal FL, with diverse multi-hospital EMR data, is addressing data distribution across sites. Weishen et al. proposed an adaptive FL framework specifically for this issue in clinical risk prediction to predict onset risk of sepsis and acute kidney injury for the patients at intense care unit (ICU). This approach separates input features and jointly learns site-specific models by selectively sharing parameters related to stable features while allowing domain specific parameters to vary [45].

While these studies highlight the progress in FL for healthcare, many FL implementations for RFs depend on simple unweighted averaging of predictions or model parameters. There is a need of systematic evaluation of more nuanced weighted average schemes that can explicitly account for different data characteristics that might potentially offer improved performance and stability over basic unweighted aggregation and dealing with real-world data.

### Objective

The primary objective of this study is to investigate whether various weighted averaging schemes for aggregating predictions from locally trained RF models can enhance performance in horizontal FL setting compared to unweighted averaging scheme.

By using large-scale, real-world EMR data from eleven hospitals for the prediction of delirium, we aimed to develop and implement FL strategies based on different weighting criteria. We systematically compared the predictive performance of these weighted FL aggregation methods against unweighted averaging, models trained on individual hospital data and general model (GM). Furthermore, we aimed to identify which methods offer advantage for RF based clinical risk prediction using multi institutional healthcare data, thereby providing insights into practical and effective FL strategies for such scenarios.

### Use-case

The classification problem addressed in this study was the identification of patients susceptible to developing delirium during hospitalisation at the time of admission. Delirium is an acute confusion state which is highly prevalent during hospitalisation, ranging from around 4% to 65% [46]. Numerous studies emphasised early identification of delirium to prevent associated complications [47, 48]. In our earlier studies, we applied RFs to predict delirium during hospitalisation using EMRs and validated the tool in healthcare routine [29, 49]. The model is currently on and running in regular use at several clinics and departments of Styrian hospitals in Austria.

## Methods

RFs consist of multiple simple and easy-to-interpret DTs [32]. FL using RF is inspired by the RFs construct itself. Since the RF is an ensemble of DTs, an ensemble of RFs is equivalent to an RF with a large set of DTs from different RFs, provided that these RFs are built with the same set of features and labels with respect to horizontal FL.

**Fig. 1:**
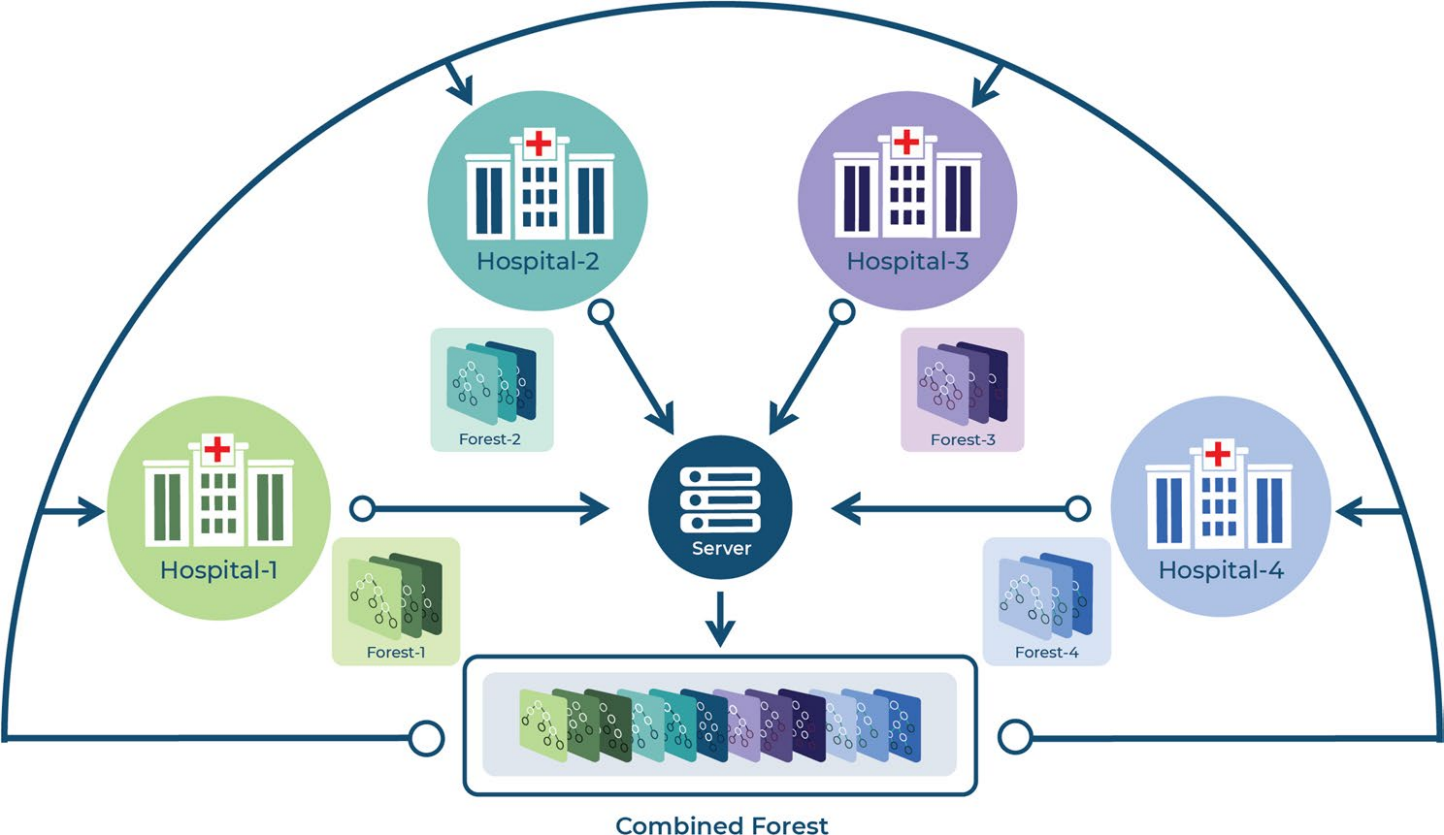
FL scheme adopted in this study. RF models were trained independently at each cooperating hospital (1–4). These individual models were transferred to a central server to be combined into a FL model. The resulting FL model was subsequently redistributed to the hospitals to improve the performance by leveraging the collective knowledge from all the participating hospitals

To establish FL using RF, our concept was to train a RF model at each hospital and transfer the individual hospital’s models to a centralised server. On the central server, the models are combined to a single FL model, which was then redistributed to the cooperating hospital as shown in Fig 1. However, the combination of models into one single RF delivers the same result as the mean of prediction probabilities of each individual RF institutional model. Thus, the mean was considered as the baseline FL. Weighted average was introduced to take the size of the hospital dataset, such as sample size, positive cases. It reflects the incidence of the disease, minority class in case if negative cases are in smaller size and the bias of the majority class that is carried by the data of each hospital into consideration.

The weighted average of the prediction probabilities is evaluated by multiplying the relative frequencies of the terminal node with weights of the individual hospitals. Summing the results from each forests’ predicting probabilities would provide FL result that corresponds to Eq. 1.

1 $$P = \mathop \sum \limits_{i = 1}^N {{\mathop \sum \nolimits_{j = 1}^{{m_i}} {p_{i,j}}} \over {{m_i}}}$$

Where $P$ is the prediction probability, $N$ is number of hospitals/institutions, ${m_i}$ is number of trees in RF of the ${i^{th}}$ hospital and ${p_{i,j}}$ is the relative frequency of the terminal node of the ${j^{th}}$ tree in the ${i^{th}}$ RF.

The following learning schemes were applied in the context of this paper.

1. Individual models, based on each hospital’s respective dataset **(“H01”-“H11”)**
2. A general model, trained on the whole dataset, without any privacy preservation and without FL **(“GM”)**
3. FL schemes
  - Without taking into account different dataset sizes: unweighted averaging, i.e., mean over results from each hospital model **(“unweighted”)**
  - Taking into account different dataset sizes:
    i) Without taking into account different levels of imbalance:
      Weighted average based on the sample size of dataset at each hospital **(“samples”)**
    ii) Taking into account different levels of imbalance:
      Weighted average based on the size of the positive samples (i.e., delirium patients) **(“positives”)**
      Weighted average based on the size of the minority number of class samples (i.e., either delirium or non-delirium patients, depending on what number was lower) **(“minority”)**
      Weighted average based on maximum possible diversity (mpd, see below) and sample size **(“mpd”)**

The weighted average based on ‘mpd’ normalises the prediction probabilities based on Shannon evenness index (SEI), accounting for class imbalance issues and avoiding bias towards the majority class [50]. The mpd was calculated according to Eq. 2.

2 $${v_i} = {E_i}*{n_i}$$

Where ${n_i}$ is number of samples of hospital $i$ and ${E_i}$ is SEI given in Eq. 3.

3 $${E_i} = {H_i}/\ln (N)$$

With $N$ representing the number of classes and ${H_i}$ the Shannon diversity index of the hospital $i$ calculated with Eq. 4.

4 $${H_i} = - \mathop \sum \limits_{j = 1}^N {\left( {{p_j}} \right)_i}\ln {\left( {{p_j}} \right)_i}$$

Where $p$ represents the proportion of class $j$ at the hospital $i$.

Equation 5 was applied to obtain the delirium probability for an individual patient as a weighted average.

5 $${\mathrm{Weighted}}\;{\mathrm{Average}} = \sum\limits_{i = 1}^n {\left( {{x_i}*{w_i}} \right)} $$

$${where}\;{w_i} = {{{v_i}} \over {\mathop \sum \nolimits_{i = 1}^n {v_i}}}$$

Where $n$ represents the number of institutions (i.e., number of RFs), ${x_i}$ represents the estimated class probability derived with the trained model of hospital $i$ and ${w_i}$ represents the weight of hospital $i$. The value ${v_i}$ varied based on the FL schemes (2–5) and represented either the total number of samples, positive, minority class samples or mpd at hospital $i$.

### Dataset

Our dataset was extracted from the hospital information system (HIS) hosted by Styrian hospital holdings (KAGes), the regional healthcare provider in Styria, which is one of the nine federal states of Austria. It contained diagnoses, interventions, medications, laboratory results, nursing assessments, demographics, and metadata of the patient movements among many others.

**Fig. 2:**
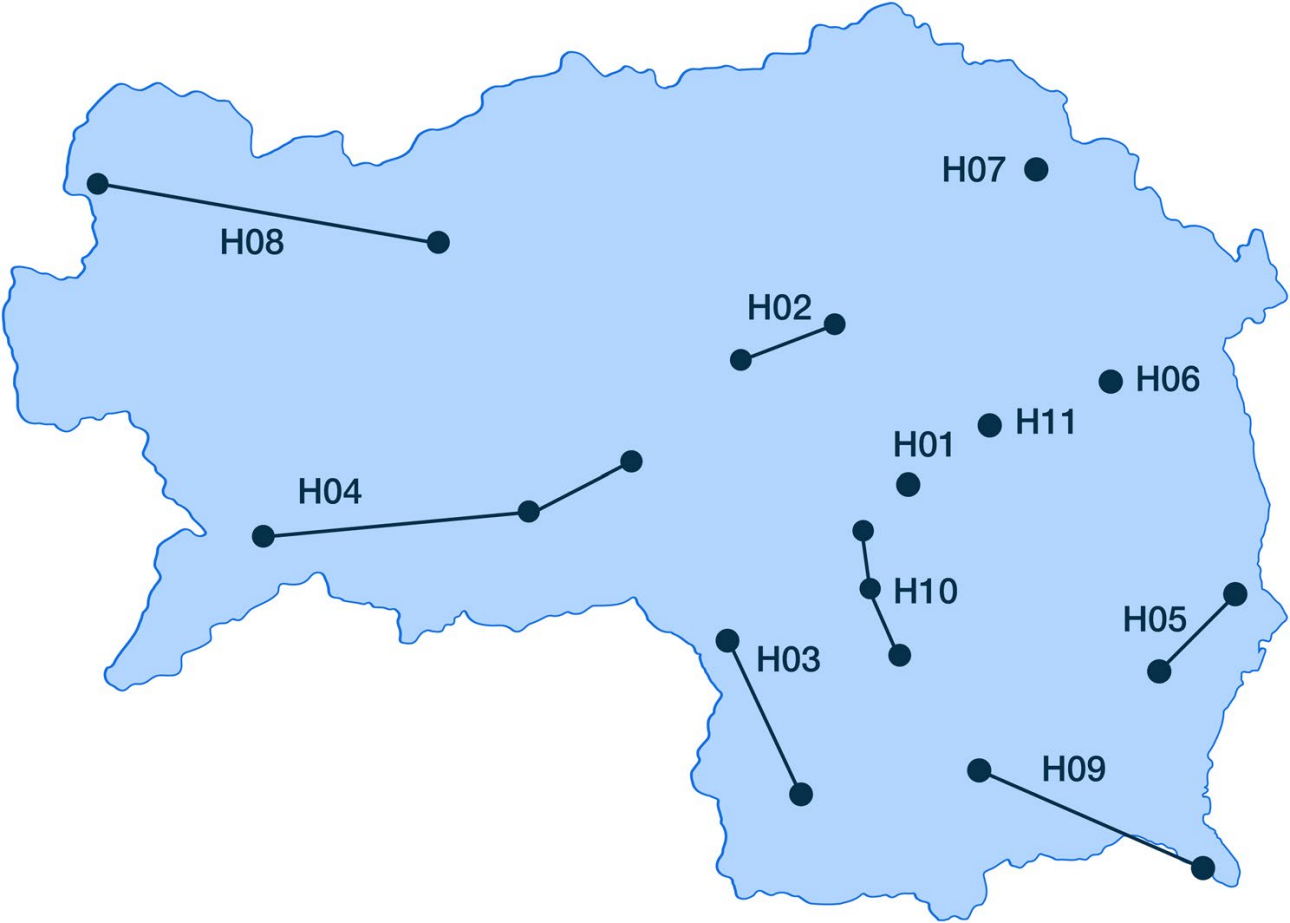
Map of the federal state Styria of Austria, indicating the locations of the hospitals

Administratively, KAGes hospitals are organised into eleven connecting networks consisting of 20 distinct hospital sites (Fig. 2). The dots in Fig 2. represent the individual physical hospitals, while the lines indicate the administrative networks that group these hospitals. For the purpose of this study, each of these eleven administrative networks was treated as separate data contributing entity, resulting in H10–H11. Although patient data from all these networks reside within the same KAGes HIS, data access and management are structured such that each network’s data can be considered logically distinct and accessed as a separate node. This organisational structure, where data from each network is effectively stored for operational purposes despite being in common system, which enables the FL approach. This grouping into eleven administrative networks therefore reflects a pragmatic approach to define FL nodes, simulating a real-world collaboration structure within the large healthcare provider system.

Our dataset consisted of a delirium cohort and a control cohort that were recorded in between the dates 2011.01.01 and 2019.03.31. The delirium cohort was identified by selecting hospitalised patients aged 18 years or older, who were diagnosed with delirium not induced by alcohol and other psychoactive substances (ICD-10 F05.*) and not diagnosed with mental and behavioural disorders due to psychoactive substances (ICD-10 F10.* – F19.*). The first delirium-diagnosed case of the patient was selected as the reference case.

The control cohort was identified by randomly selecting reference cases of patients aged 18 years or older from internal medicine and surgical departments without a delirium diagnosis and not included in the delirium cohort. Since each hospital has specific specialisations, we have concluded up on 20% of incidence all over KAGes hospitalisations in patients above 18 years old. Thus, 80% of controls were selected randomly from internal medicine department where the majority of delirium cases were documented. The criteria was to select a hospitalised case and exclude the respective patient from the selection.

**Fig. 3:**
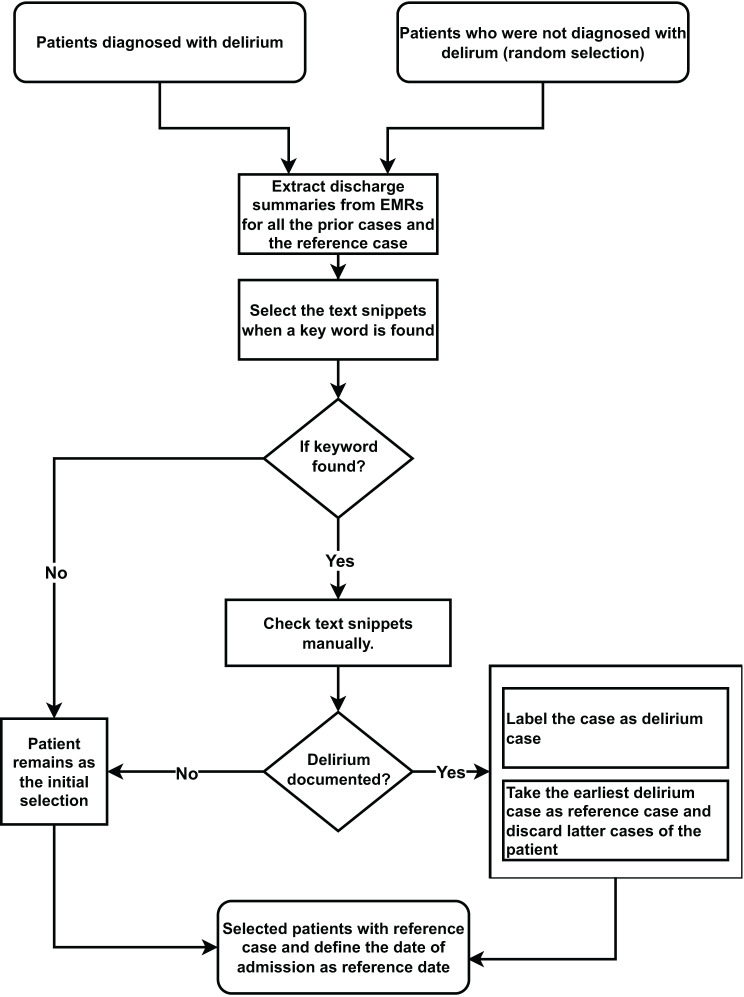
Process of re-defining the target variable delirium

To reflect a real-world FL setup, we identified the hospital where the selected reference case of both cohort and control was registered and extracted the clinical history of these cases only from the corresponding hospital. Since delirium is often not ICD-10 coded, we collected the discharge summaries for both delirium and control patients corresponding to all the hospitalised cases prior to the reference case and manually corrected falsely labelled cases by identifying certain keywords “brief psychotic disorder”, “acute confusion”, “passive confusion” and “delirium“in the discharge summaries (see Fig. 3). The labels of the texts were annotated by two data scientists who are native German speakers.

Finally, 29,479 patients were included in the study cohort, whereas 8900 were delirium and 20,579 were non-delirium patients. The control cases were selected randomly three times bigger than the size of the delirium cohort. However, after re-labelling, a few cases were added to delirium cohort. Detailed patient characteristics at each hospital are depicted in Table 1.

**Table 1:** An overview of patients’ characteristics including age, gender, diagnoses associated to delirium, number of delirium cases and total number of samples per hospital

	H01	H02	H03	H04	H05	H06	H07	H08	H09	H10	H11
Age [years] (mean ± standard deviation)	63.10 ± 17.10	67.20 ± 17.00	68.50 ± 18.00	64.10 ± 18.00	66.9 0 ± 19.70	64.70 ± 17.70	70.40 ± 18.00	68.20 ± 18.20	66.10 ± 17.50	71.10 ± 16.70	65.30 ± 19.10
**Gender (n)**											
Male	2,790	2,354	905	1,354	1,085	641	252	879	1,017	3,580	388
Female	2,243	2,014	1,013	1,550	1,075	630	300	994	1,019	3,044	352
**Target variable (simulated incidence)**											
Observed Delirium	953 (18.9%)	1,000 (22.9%)	390 (20.3%)	408 (14%)	385 (17.8%)	287 (22.6%)	160 (29%)	628 (33.5%)	408 (20%)	4,197 (63.4%)	84 (11.4%)
**Previously coded diagnoses in patient history**											
Dementia, Alzheimer	51 (1%)	127 (2.9%)	84 (4.4%)	100 (3.4%)	97 (4.5%)	59 (4.6%)	24 (4.3%)	117 (6.2%)	61 (3%)	313 (4.7%)	19 (2.6%)
Parkinson Disease	49 (1%)	55 (1.3%)	40 (2.1%)	49 (1.7%)	60 (2.8%)	27 (2.1%)	12 (2.2%)	78 (4.2%)	21 (1%)	103 (1.6%)	7 (0.9%)
Depression	190 (3.8%)	266 (6.1%)	140 (7.3%)	267 (9.2%)	148 (6.9%)	100 (7.9%)	44 (8%)	289 (15.4%)	122 (6%)	590 (8.9%)	38 (5.1%)
Cerebral infarction, Stroke	132 (2.6%)	106 (2.4%)	51 (2.7%)	130 (4.5%)	96 (4.4%)	34 (2.7%)	4 (0.7%)	59 (3.2%)	38 (1.9%)	154 (2.3%)	12 (1.6%)
Cerebrovascular diseases	406 (8.1%)	288( 6.6%)	157 (8.2%)	246 (8.5%)	212 (9.8%)	68 (5.4%)	70 (12.7%)	267 (14.3%)	132 (6.5%)	347 (5.2%)	40 (5.4%)
Epilepsy and recurrent seizures	79 (1.6%)	106 (2.4%)	42 (2.2%)	68 (2.3%)	76 (3.5%)	28 (2.2%)	9 (1.6%)	48 (2.6%)	37 (1.8%)	175 (2.6%)	9 (1.2%)
Alcohol related disorders/Alcoholic liver disease	8 (1.7%)	136 (3.1%)	68 (3.5%)	82 (2.8%)	72 (3.3%)	50 (3.9%)	27 (4.9%)	86 (4.6%)	66 (3.2%)	510 (7.7%)	18 (2.4%)
Volume depletion or other fluid disorders of, electrolyte and acid-base balance	108 (2.1%)	124 (2.8%)	61 (3.2%)	92 (3.2%)	83 (3.8%)	19 (1.5%)	31 (5.6%)	114 (6.1%)	50 (2.5%)	165 (2.5%)	43 (5.8%)
Number of cases	**5,033**	**4,368**	**1,918**	**2,904**	**2,160**	**1,271**	**552**	**1,873**	**2,036**	**6,624**	**740**

Sex distribution was almost equal across all hospitals. The incidence of delirium varied between 11.40% and 63.40% across the different hospitals. Notably, the hospital with a specialisation in geriatric patients had the highest incidence of delirium. The mean age of the patients varied between 63.10 and 71.10 years. The relative share of associated comorbidities varied slightly across hospitals, which can be attributed to differences in specialisations offered by each hospital.

### Features

All features were derived from EMR prior to the reference case at the hospital it was recorded. The following categories of features were analysed.

- Demographics (e.g. age, gender, etc.)
- Diagnoses: Relevant ICD-10 diagnoses were selected based on bivariate statistical association. The Charlson Comorbidity Index was calculated from the recorded diagnoses. Suspected diagnoses that have not been confirmed in the KAGes HIS at hospital discharge by the attending physician were not considered.
- Procedures documented according to the Austrian diagnosis-related group (DRG) System. Procedures recorded in less than ten cases were excluded. Selected procedures were aggregated to their group level.
- Administrative data concerning the patient stay at hospital.
- Laboratory results
- Nursing assessments
- Medications

ICD-10 codes, procedural codes, laboratory value abnormalities, gender, nursing assessments, and medications, were considered as categorical data and either one-hot-encoding or multi-hot-encoding was applied. Age, length of longest hospitalized stay, etc. were considered as numerical values. Table 2 provides an overview of all feature groups.

**Table 2:** List of feature categories with few examples and number of features per category

Feature category	Examples	n
Demographics	Age, gender	11
Diagnoses	ICD codes, ICD groups	170
Procedures	ICPM codes	80
Transportation	Ambulance, by foot	12
Laboratory	Gamma GT, sodium	108
Nursing Assessment	Oriented, fall	93
Medication	ATC codes	122
Counts	Number of admissions/hospitalisations	17
Location	Postal codes	14
**Total**		**627**

### Training and testing

We separated the dataset into eleven parts based on the hospitals where the reference case was registered. We applied a stratified 5-fold cross validation (CV) framework to separate training and test datasets to capture the variance of model performance between five folds.

To identify the optimal hyper-parameters for the RF models—namely, the number of trees (ntree) and the number of features for each split (mtry)—we employed a nested approach: a 5-repeated 10-fold CV was used for hyper-parameter tuning within the training folds, while outer 5-fold CV was used for final testing of the RF models. Grid search over the tuning range for ntree (100, 200, 300, 400, 500, 600, 700, 800, 900, 1000) and mtry (1:30) was applied. Accuracy was used as the optimisation metric to find the optimal hyper-parameters. A list of hyper-parameters per fold per model has been tabulated in supplementary materials Table 1.

**Fig. 4:**
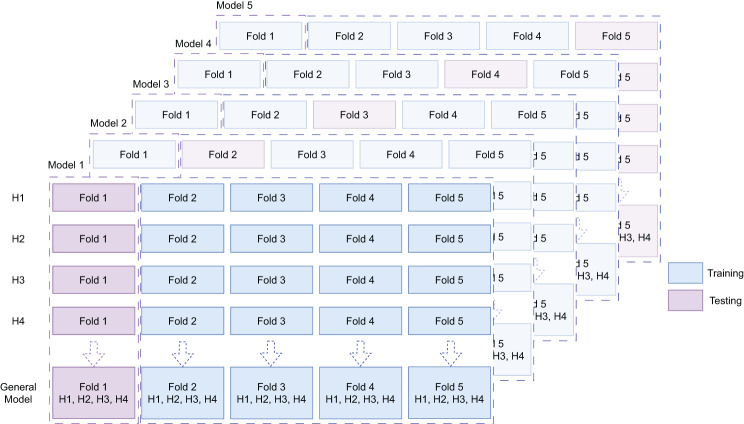
5-fold CV was applied for training and testing the models. The folds derived for the models per hospital (illustrated for H01–H04) were also applied for training and testing the general model (GM)

Each hospital data was split into five equal parts and named as $Fold\;1 - Fold\;5$. We defined $I = \left\{ {1,2,3,4,5} \right\}$, K= {H01, H02, H03, H04, H05, H06, H07, H08, H09, H10, H11}, $i,j \in I$ and $k \in K,$where $i$ and $j$ are the indices of folds and $k$ is a hospital. For each hospital $k$, we took $dat{a_{k,i}}$ (i.e. 20%) as the test set and $\mathop \cup \limits_{j \in I, j \ne i} \left( {dat{a_{k,j \ne i}}} \right)$(i.e, 80%) as training set. For the GM we took $\mathop \cup \limits_{k \in K} \left( {dat{a_{k,i}}} \right)$ as the test set and $\mathop \cup \limits_{k \in K,j \in I,j \ne i} \left( {dat{a_{k,j \ne i}}} \right)$ as the training set (see Fig. 4). Thus, hyper-parameter tuning was confined strictly to the training data and test folds remained completely unseen until final evaluation. In this way, four parts of the data were used to train (with internal validation for tuning) and one part was used for testing, resulting in five models and five test sets per hospital as well as for GM.

In our framework, the inner CV was used solely for hyper-parameter tuning within the training data, while the outer CV was reserved for model evaluation. This ensures that hyper-parameters are optimised without leaking information from the test folds, and that the outer CV provides an unbiased estimate of model variability across folds.

The receiver operating characteristic (ROC) and area under the ROC (AUROC) were considered as the performance measure. The AUROC provides a summary measure of the model’s performance across various thresholds and it is used as a benchmark for evaluating the discriminative power of ML models [51]. Table 3 contains sensitivity and specificity of each model calculated at the threshold determined at the maximum “Youden” index.

**Table 3:** The mean and 95% confidence interval of the AUROC of each scheme tested with the “combined test data” along with sensitivity and specificity calculated at the threshold determined at the maximum “Youden” index

MODEL	Mean AUROC	95% Confidence Interval	Sensitivity	Specificity
H01	0.800	[0.788–0.811]	0.802 (0.020)	0.657 (0.033)
H02	0.742	[0.729–0.756]	0.736 (0.023)	0.633 (0.034)
H03	0.722	[0.708–0.735]	0.720 (0.054)	0.613 (0.039)
H04	0.734	[0.720–0.747]	0.736 (0.037)	0.622 (0.038)
H05	0.753	[0.741–0.766]	0.790 (0.024)	0.603 (0.044)
H06	0.734	[0.721–0.748]	0.748 (0.063)	0.601 (0.035)
H07	0.678	[0.663–0.693]	0.610 (0.091)	0.677 (0.050)
H08	0.733	[0.719–0.746]	0.712 (0.033)	0.653 (0.048)
H09	0.684	[0.670–0.698]	0.815 (0.046)	0.454 (0.057)
H10	0.829	[0.818–0.841]	0.721 (0.025)	0.774 (0.018)
H11	0.633	[0.618–0.649]	0.657 (0.031)	0.541 (0.034)
General Model (GM)	0.855	[0.845–0.865]	0.757 (0.028)	0.780 (0.035)
unweighted	0.794	[0.782–0.806]	0.772 (0.029)	0.676 (0.032)
samples	0.830	[0.819–0.841]	0.782 (0.019)	0.723 (0.011)
positives	0.843	[0.832–0.854]	0.760 (0.020)	0.761 (0.017)
minority	0.841	[0.830–0.852]	0.773 (0.014)	0.749 (0.009)
mpd	0.836	[0.825–0.847]	0.774 (0.020)	0.743 (0.024)

The statistical software R was used to extract the data from HIS, perform data wrangling and apply ML algorithms. The R packages that were used included caret, odbc, ggplot2, randomForest and the tidyverse package bundle. 

## Results

### Dataset characteristics

The final dataset used in this study included 29,479 patients with 627 features. Table 4 summarises the number of samples per hospital along with the number of positive, minority class, and associated proportion of mpd.

**Table 4:** List of number of samples, positives, minority classes, and mpd as well as corresponding weights for each scheme per hospital H01–H11. v represents the value and w represents the weight calculated using Eq. 4

	samples		positives		minority		mpd	
	$v$	$w$	$v$	$w$	$v$	$w$	$v$	$w$
H01	5033	0.171	953	0.107	953	0.134	3523.655	0.154
H02	4368	0.148	1000	0.112	1000	0.140	3390.244	0.149
H03	1918	0.065	390	0.044	390	0.055	1397.362	0.061
H04	2904	0.099	408	0.046	408	0.057	1700.396	0.074
H05	2160	0.073	385	0.043	385	0.054	1460.620	0.064
H06	1271	0.043	287	0.032	287	0.040	979.469	0.042
H07	552	0.019	160	0.018	160	0.022	479.430	0.021
H08	1873	0.064	628	0.071	628	0.088	1723.611	0.075
H09	2036	0.069	408	0.046	408	0.057	1471.444	0.064
H10	6624	0.225	4197	0.472	2427	0.340	6278.649	0.275
H11	740	0.025	84	0.009	84	0.012	377.713	0.016
**Total**	**29479**	**1.000**	**8900**	**1.000**	**7130**	**1.000**	**22782.6**	**1.000**

**Fig. 5:**
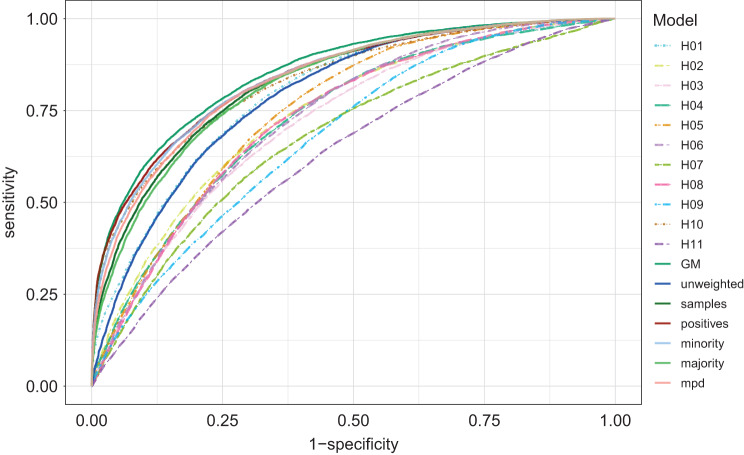
ROC of all the models tested with the combined test data ($${ \cup _{{\mathrm{k}} \in {\mathrm{K}}}}(dat{a_{{\mathrm{k}},{\mathrm{i}}}})$$ $$)$$. Dashed lines represent the individual hospitals’ model; solid lines represent the general model (GM) and FL models

Figure 5 shows the ROC curves of all models. Table 3 present the mean AUROCs and 95% confidence intervals of AUROC across five folds.

- The GM, which was trained with data from all hospitals, outperformed all the other models with an AUROC of 0.855 [0.845-0.865].
- Among FL models, averaging with weights based on the number of positive cases (delirium patients) performed the best, with an AUROC of 0.843 [0.832-0.854], followed closely by minority class 0.841 [0.830-0.852].
- The unweighted FL model performed better than most individual hospital models (AUROC 0.794 [0.782-0.806]), but worse than weighted schemes.

Hospital-specific models, performed worse than the FL schemes. H01 and H10, which were the hospitals with the highest number of patients, were closest to the best FL models with AUROCs of 0.800 [0.788–0.811] and 0.829 [0.818–0.841], respectively.

**Fig. 6:**
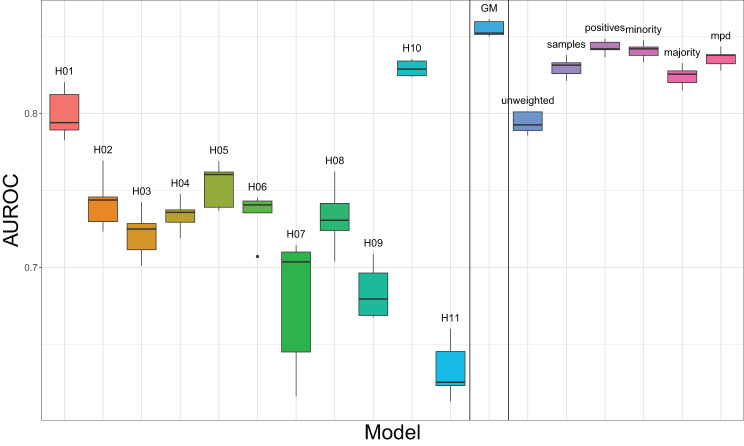
Boxplots of AUROC tested with combined test data

The boxplots in Fig 6 show the variance of the models achieved from the five folds of each hospital’s models, the GMs and each of the FL schemes.

Table 5 compares AUROC obtained from testing each single hospital’s test data by its own model, GM and different FL schemes. This highlights that, the small hospitals benefited the most from FL aggregation, while large hospitals achieved strong performance with their own models.

**Table 5:** The performance of the individual hospitals’ models when applied on their own dataset (“own”) was compared to the performance, when the other models (general, unweighted, samples, positives, minority or mpd) were applied on the same datasets

Test data	own model	general	unweighted	samples	positives	minority	mpd
H01	0.776 [0.76–0.792]	0.778 [0.764–0.792]	0.734 [0.715–0.752]	0.756 [0.738–0.774]	0.750 [0.732–0.767]	0.756 [0.738–0.773]	0.757 [0.739–0.775]
H02	0.760 [0.742–0.778]	0.759 [0.749–0.768]	0.748 [0.733–0.763]	0.755 [0.742–0.768]	0.746 [0.735–0.756]	0.753 [0.742–0.765]	0.755 [0.743–0.768]
H03	0.737 [0.705–0.768]	0.766 [0.74–0.791]	0.749 [0.722–0.776]	0.755 [0.73–0.779]	0.754 [0.733–0.774]	0.757 [0.734–0.779]	0.756 [0.733–0.78]
H04	0.834 [0.81–0.858]	0.843 [0.827–0.858]	0.851 [0.826–0.876]	0.856 [0.835–0.877]	0.841 [0.82–0.861]	0.852 [0.831–0.873]	0.855 [0.833–0.876]
H05	0.752 [0.743–0.762]	0.783 [0.772–0.793]	0.741 [0.727–0.755]	0.758 [0.745–0.771]	0.763 [0.745–0.781]	0.764 [0.749–0.779]	0.761 [0.747–0.775]
H06	0.761 [0.742–0.781]	0.772 [0.756–0.789]	0.755 [0.732–0.777]	0.756 [0.734–0.778]	0.761 [0.743–0.78]	0.761 [0.741–0.781]	0.758 [0.737–0.779]
H07	0.699 [0.642–0.756]	0.682 [0.603–0.761]	0.683 [0.628–0.738]	0.674 [0.616–0.732]	0.669 [0.605–0.733]	0.675 [0.614–0.735]	0.676 [0.617–0.735]
H08	0.795 [0.765–0.825]	0.774 [0.74–0.809]	0.773 [0.75–0.795]	0.773 [0.746–0.799]	0.765 [0.733–0.798]	0.774 [0.744–0.804]	0.774 [0.745–0.803]
H09	0.777 [0.76–0.794]	0.798 [0.779–0.817]	0.785 [0.768–0.803]	0.790 [0.772–0.808]	0.794 [0.775–0.813]	0.793 [0.775–0.812]	0.792 [0.774–0.81]
H10	0.911 [0.905–0.917]	0.908 [0.902–0.914]	0.831 [0.818–0.844]	0.889 0.877–0.901]	0.915 [0.908–0.923]	0.908 [0.898–0.917]	0.899 [0.888–0.91]
H11	0.718 [0.653–0.783]	0.775 [0.717–0.833]	0.754 [0.684–0.824]	0.761 [0.698–0.824]	0.780 [0.729–0.831]	0.773 [0.718–0.827]	0.766 [0.706–0.825]

## Discussion

### Key findings and interpretation

This study investigated the efficacy of different FL prediction aggregation schemes for RF models in predicting delirium, using real-world EMR datasets from eleven Austrian hospital networks. Our primary finding is that FL models, particularly those employing weighted averaging schemes based on site-specific data characteristics, can achieve robust predictive performance, closely approaching the performance of a GM and significantly outperforming most individual hospital models and simple unweighted averaging FL scheme.

As anticipated, the model trained with the all pooled training dataset i.e. GM outperformed all hospital specific models, yielding the highest performance (AUROC 0.855 [0.845–0.865] with a sensitivity of 0.757 and specificity of 0.780) (Table 3), serving as an important benchmark. The simplest FL model, which averaged predictions derived from all hospital-specific models i.e. unweighted model in our case, outperformed many individual hospital models by achieving an AUROC of 0.794 but was notably outperformed by weighted averaging schemes. The positives (AUROC 0.843 [0.832–0.854] with a sensitivity of 0.760 and specificity of 0.761) and minority (AUROC 0.841 [0.830–0852] with a sensitivity of 0.773 and specificity of 0.749) weighted schemes performed best, achieving approximately 98.6% and 98.4% of the GM’s AUROC respectively. These findings highlight that FL can be a highly effective, privacy-preserving alternative with only marginal performance loss. This outcome is comparable to, or even exceeds, performance expectations from other FL studies. The clear superiority of weighted schemes over unweighted averaging underscores the value of incorporating site-specific data characteristics into the FL aggregation process when dealing with heterogeneous data.

The models from the largest hospitals H01 (AUROC 0.800 [0.788–0.811]) and H10 (AUROC 0.829 [0.818–0.841]) performed better than the unweighted model and in general performed better locally. These results can be explained by the poor performance of those models trained with very small datasets (e.g., H11), which contribute equally to the unweighted average. Therefore, the unweighted averaging model could be valuable for small and medium sized hospitals. However, for large hospitals, training of individual models with their own data only would be preferred for deployment.

Unweighted averaging is considered as a valid method, when distributed datasets have similar sizes and class imbalances. For datasets of different sizes but similar level of class imbalance, weights based on the sample size are recommended. If all datasets have the same class as majority samples, weighting based on positive cases should be considered. Since this was the case for all but one hospital, minority class and positive cases showed very similar results in our study. Moreover, our approach based on mpd, while showing slightly worse results compared to positives and minority, could be applied for multi-class classification problems, unlike other averaging methods that are limited to two classes. From the results (see Table 5), we interpret that small hospitals benefited the most from our federated models. For large hospitals, it may be better to use models trained only on their own data because they have sufficient data for training and the model can fit well to the hospitals’ patient characteristics. However, relying only on one owns’ data can lead to less stable models, particularly for a type of patient who have not been admitted to the hospital previously.

A key practical advantage of the FL models demonstrated in this study is their enhanced consistency. Figure 6 demonstrates that, FL models have smaller interquartile ranges than individual hospital models, indicating more consistence in their predictions. Therefore, there is a significant risk of distributing inferior models to various hospitals if individual, non-FL models were used.

### Clinical utility

Models achieving AUROCs around 0.84–0.85, such as the GM and the positives FL scheme, are clinically relevant. With sensitivities around 0.76 and specificities ranging from 0.76 to 0.78, these models can correctly identify approximately three-quarters of both delirium and non-delirium cases. Given the serious consequences of undiagnosed delirium, this level of sensitivity is clinically relevant.

Individual hospital models with more modest performance, like H11 (AUROC 0.633 [0.618–0.649] with a sensitivity of 0.657 and specificity of 0.541) or H09 (AUROC 0.684 [0.670–0.698] with a sensitivity of 0.815 and specificity of 0.454 – noting its poor specificity), their standalone utility is limited, but they highlight the baseline from which FL offers improvement. For H09, an FL model like positives (Table 5: AUROC 0.794 [0.775–0.813] for H09 data) would likely offer a better diagnostic balance.

### Comparison with other methods

RFs were chosen due to their robustness and prior superior performance in delirium prediction compared to ANNs [29]. Although ANNs are widely applied successfully in many clinical scenarios, they have several disadvantages in terms of computational costs, interpretability and ability to explain the results.

While there is a lot of research ongoing in the field of FL based on ANNs, our study demonstrates that RFs can be a valuable and computationally efficient alternative for FL in healthcare with a simple weighted averaging.

### Strengths

We used real world data extracted from eleven hospitals to demonstrate our approaches. Whereas, Hauschild et al. [38] used datasets with limited sample sizes. Yang Liu et al. [33] in their paper Federated Forest, discussed the vertical FL where they are using the same observations with different feature space. In our case, it is horizontal FL, where the sample space is different and the feature space is the same. A delirium prediction model is already deployed in clinical routine and prospectively validated [29, 49] that was developed using this real-world EMR data. Thus, our study provides distinct insights compared to studies using benchmark datasets.

Future work could explore aggregating feature importance from local models in an FL setting, potentially using similar weighting schemes, to enhance global explainability.

### Limitations

The dataset utilised in this study exhibits class imbalance and comprised data from hospitals representing diverse medical disciplines, including a large university clinic (H02) and a specialised geriatrics hospital (H10). Although the performance of all the FL schemes closely approached either the GM or the respective individual hospitals’ models, the confidence interval of AUROC values overlapped, indicating that the observed improvement is not statistically significant. However, a clear trend of better results using FL could be identified.

Additionally, we have not yet analysed, how some of the features of RFs, such as feature importance measurement, can be applied on the FL approaches. Up to now, our methods have been tested on one specific use case, i.e., prediction of delirium. Further research will be necessary to clarify whether similar results can be achieved with different types of data, different dataset sizes, different degrees of imbalance, etc. Finally, the whole setting of our study was retrospective, and no prospective validation was applied. While 5-fold CV was appropriate for our objectives of comparing multiple methods and assessing performance variance, a final prospective validation or evaluation on a completely distinct, held-out test set using a strict train/validation/test split would be standard for assessing a single, finalized deployment model or for direct comparison against SOTA on a public benchmark. Due to the sensitive nature of our multi-hospital EMR data and the lack of directly comparable public EMR benchmarks for delirium prediction with this feature set, direct comparison on such a benchmark was not feasible. The definition of hospital nodes based on administrative networks was a logical choice reflecting real-world collaboration structures.

### Outlook

While we applied weighted averaging for combining prediction probabilities of RFs, future studies could directly combine the trees of the individual forests into a single RF to exploit some more advantages of RFs, such as global feature importance. However, this would require the development of suitable and sustainable distributed models for such approaches, and further studies will be required to explore their potential benefits in the healthcare field. Randomly removing the trees before calculating prediction probabilities would a point to add. Feature separation with our weighted averaging schemes could also be a fruitful direction.

Future work will also include the application of our approach for applications other than delirium prediction. Finally, a prospective study on applying the developed FL scheme is required. To increase further privacy, introducing a privacy layer would add a value.

## Conclusions

In this paper, we systematically evaluated five federated learning schemes for random forests to predict delirium patients using EMRs from eleven hospitals. Our empirical study found that the proposed federated learning methods achieved comparable performance to general models, which highlights their potential to solve the problem of training generalised machine learning models without sharing data. Furthermore, results showed that the weighted averaging of random forests trained at individual hospitals outperformed both individual hospital models and unweighted averaging. These findings suggest that federated learning using random forest can be a valuable tool in healthcare for improving patient outcome while protecting patient privacy [52].

## Electronic supplementary material

Below is the link to the electronic supplementary material.

Supplementary Material 1

## Data Availability

The dataset used in this study are not publically available for sharing due to confidentiality agreements and ethical considerations. We understand that data sharing is important for transparency and reproducibility and we regret any inconvenience this may cause. However, we are willing to collaborate with other researchers who are interested in applying algorithms to our data. Please contact the corresponding author. The functional code used to generate the results in this paper is available at [https://gitlab.com/sai.veeranki/flrandomforest].

## Abbreviations

AI: Artificial Intelligence
ANN: Artificial Neural Networks
ATC: The Anatomical Therapeutic Chemical
AUROC: Area Under Receiver Operating Characteristic
CV: Cross Validation
DT: Decision Tree
FL: Federated Learning
GM: General Model
GBDT: Gradient Boosting Decision Tree
HIS: Hospital Information System
ICD-10: International Classification of Diseases, Tenth revision
ICPM: International Classification of Procedures in Medicine
ML: Machine Learning
mpd: Maximum possible diversity
RF: Random Forest
ROC: Receiver Operating Characteristic
SEI: Shannon Evenness Index
