# Supplementary material for: Development and validation of random-forest based federated ensemble learning algorithms for delirium prediction using electronic medical records from eleven hospitals in Austria: a retrospective study
Source: BMC Med Inform Decis Mak. 2026 Jan 14;26:41. doi: 10.1186/s12911-025-03322-y (PMC12888642; doi:10.1186/s12911-025-03322-y)
Supplement: Supplementary file 1 — Supplementary Material 1 [file 12911_2025_3322_MOESM1_ESM.docx]

Supplementary material

# Description

This supplementary material consist of the tables and figures that provide the reader with additional information about the performance of each hospital model on different test datasets.

Supplementary table 1 shows the optimised hyper-parameters used for the training of the models.

Supplementary table 2 presents the mean accuracy, sensitivity, specificity, precision and F1 score over five folds metrics when tested with combined test dataset at the optimum threshold selected at the maximum Youden index.

Supplementary table 3-13 provides the information presented in Table 2 of the manuscript, when the models tested with all the hospitals test data. In these tables, we have added an aditional FL scheme called majority which was also part of the investigation and strategically not included in the manuscript.

Additionally, the supplementary Fig S1 – S11 show the box plots that represent the performance of the models when tested with individual hospitals’ test data.

The heatmap in Fig S12 shows the perfomance of all the models tested with all sets of test data. This will give the reader an overview of how every model is peforming on other hospitals’ test data as well as the combined dataset.

Supplementary table 1 Optimised parameters from hyper-parameter optimisation

| FOLD | PARAMS | GM | H01 | H02 | H03 | H04 | H05 | H06 | H07 | H08 | H09 | H10 | H11 |
| --- | --- | --- | --- | --- | --- | --- | --- | --- | --- | --- | --- | --- | --- |
| Fold1 | mtry | 27 | 30 | 30 | 29 | 19 | 7 | 7 | 30 | 29 | 29 | 29 | 10 |
| Fold1 | ntree | 1000 | 700 | 600 | 700 | 300 | 100 | 100 | 600 | 900 | 200 | 600 | 100 |
| Fold1 | Accuracy | 0.81 | 0.81 | 0.78 | 0.80 | 0.86 | 0.822 | 0.78 | 0.74 | 0.74 | 0.80 | 0.85 | 0.89 |
| Fold2 | mtry | 30 | 30 | 30 | 15 | 30 | 6 | 7 | 30 | 30 | 25 | 26 | 10 |
| Fold2 | ntree | 800 | 600 | 200 | 200 | 1000 | 100 | 300 | 1000 | 600 | 100 | 900 | 100 |
| Fold2 | Accuracy | 0.81 | 0.82 | 0.78 | 0.80 | 0.86 | 0.82 | 0.80 | 0.71 | 0.75 | 0.81 | 0.87 | 0.89 |
| Fold3 | mtry | 24 | 29 | 26 | 29 | 28 | 4 | 30 | 30 | 28 | 20 | 29 | 12 |
| Fold3 | ntree | 1000 | 400 | 200 | 600 | 300 | 100 | 300 | 600 | 700 | 600 | 1000 | 100 |
| Fold3 | Accuracy | 0.80 | 0.82 | 0.78 | 0.80 | 0.86 | 0.82 | 0.80 | 0.73 | 0.74 | 0.80 | 0.85 | 0.89 |
| Fold4 | mtry | 30 | 23 | 29 | 27 | 30 | 7 | 30 | 29 | 30 | 30 | 30 | 18 |
| Fold4 | ntree | 700 | 700 | 800 | 700 | 500 | 300 | 300 | 200 | 700 | 1000 | 600 | 200 |
| Fold4 | Accuracy | 0.80 | 0.82 | 0.78 | 0.80 | 0.86 | 0.82 | 0.80 | 0.72 | 0.74 | 0.80 | 0.85 | 0.89 |
| Fold5 | mtry | 28 | 30 | 30 | 29 | 22 | 5 | 29 | 30 | 30 | 30 | 30 | 26 |
| Fold5 | ntree | 600 | 200 | 100 | 700 | 700 | 100 | 600 | 600 | 600 | 1000 | 700 | 100 |
| Fold5 | Accuracy | 0.81 | 0.82 | 0.78 | 0.80 | 0.86 | 0.82 | 0.80 | 0.73 | 0.74 | 0.80 | 0.859 | 0.89 |

Supplementary table 2 Mean of accuracy, sensitivity, specificity, precision, and F1 score of all the models tested with the combined test dataset over 5 folds with standard deviation at the optimum threshold selected at maximum Youden index

| Model | Accuracy | Sensitivity | Specificity | Precision | F1 |
| --- | --- | --- | --- | --- | --- |
| H01 | 0.701 (0.020) | 0.802 (0.020) | 0.657 (0.033) | 0.504 (0.022) | 0.618 (0.015) |
| H02 | 0.664 (0.021) | 0.736 (0.023) | 0.633 (0.034) | 0.465 (0.020) | 0.570 (0.015) |
| H03 | 0.645 (0.013) | 0.720 (0.054) | 0.613 (0.039) | 0.446 (0.010) | 0.550 (0.014) |
| H04 | 0.657 (0.017) | 0.736 (0.037) | 0.622 (0.038) | 0.458 (0.014) | 0.564 (0.008) |
| H05 | 0.659 (0.024) | 0.790 (0.024) | 0.603 (0.044) | 0.464 (0.020) | 0.584 (0.011) |
| H06 | 0.646 (0.008) | 0.748 (0.063) | 0.601 (0.035) | 0.448 (0.006) | 0.560 (0.019) |
| H07 | 0.657 (0.026) | 0.610 (0.091) | 0.677 (0.050) | 0.450 (0.029) | 0.516 (0.047) |
| H08 | 0.671 (0.025) | 0.712 (0.033) | 0.653 (0.048) | 0.472 (0.027) | 0.567 (0.014) |
| H09 | 0.563 (0.029) | 0.815 (0.046) | 0.454 (0.057) | 0.393 (0.017) | 0.530 (0.014) |
| H10 | 0.758 (0.007) | 0.721 (0.025) | 0.774 (0.018) | 0.580 (0.012) | 0.643 (0.006) |
| H11 | 0.576 (0.019) | 0.657 (0.031) | 0.541 (0.034) | 0.383 (0.013) | 0.484 (0.013) |
| GM | 0.773 (0.016) | 0.757 (0.028) | 0.780 (0.035) | 0.600 (0.028) | 0.668 (0.008) |
| unweighted | 0.705 (0.016) | 0.772 (0.029) | 0.676 (0.032) | 0.508 (0.017) | 0.613 (0.010) |
| samples | 0.741 (0.007) | 0.782 (0.019) | 0.723 (0.011) | 0.550 (0.009) | 0.646 (0.009) |
| positives | 0.760 (0.007) | 0.760 (0.020) | 0.761 (0.017) | 0.579 (0.011) | 0.657 (0.005) |
| minority | 0.756 (0.005) | 0.773 (0.014) | 0.749 (0.009) | 0.571 (0.006) | 0.657 (0.006) |
| majority | 0.731 (0.018) | 0.792 (0.03) | 0.704 (0.037) | 0.538 (0.024) | 0.64 (0.01) |
| mpd | 0.752 (0.013) | 0.774 (0.020) | 0.743 (0.024) | 0.567 (0.019) | 0.654 (0.010) |

Supplementary table 3 Mean of accuracy, sensitivity, specificity, precision, and F1 score of all the models tested with the H01 test dataset over 5 folds with standard deviation at the optimum threshold selected at maximum Youden index

| Model | Accuracy | Sensitivity | Specificity | Precision | F1 |
| --- | --- | --- | --- | --- | --- |
| H01 | 0.676 (0.025) | 0.802 (0.038) | 0.647 (0.035) | 0.347 (0.03) | 0.484 (0.029) |
| H02 | 0.711 (0.039) | 0.626 (0.086) | 0.732 (0.065) | 0.358 (0.053) | 0.45 (0.036) |
| H03 | 0.563 (0.085) | 0.759 (0.109) | 0.519 (0.126) | 0.274 (0.038) | 0.398 (0.028) |
| H04 | 0.565 (0.042) | 0.732 (0.062) | 0.526 (0.064) | 0.266 (0.015) | 0.389 (0.014) |
| H05 | 0.604 (0.065) | 0.773 (0.079) | 0.564 (0.101) | 0.298 (0.035) | 0.427 (0.025) |
| H06 | 0.574 (0.056) | 0.726 (0.1) | 0.539 (0.09) | 0.271 (0.029) | 0.392 (0.021) |
| H07 | 0.624 (0.073) | 0.644 (0.062) | 0.619 (0.105) | 0.291 (0.048) | 0.397 (0.032) |
| H08 | 0.66 (0.068) | 0.651 (0.115) | 0.664 (0.107) | 0.319 (0.048) | 0.421 (0.03) |
| H09 | 0.591 (0.083) | 0.671 (0.163) | 0.576 (0.143) | 0.277 (0.044) | 0.38 (0.009) |
| H10 | 0.685 (0.029) | 0.671 (0.038) | 0.688 (0.042) | 0.335 (0.024) | 0.447 (0.024) |
| H11 | 0.492 (0.095) | 0.744 (0.135) | 0.434 (0.147) | 0.238 (0.027) | 0.356 (0.021) |
| GM | 0.683 (0.037) | 0.791 (0.075) | 0.659 (0.061) | 0.354 (0.038) | 0.486 (0.027) |
| unweighted | 0.643 (0.048) | 0.766 (0.081) | 0.616 (0.072) | 0.32 (0.036) | 0.448 (0.03) |
| samples | 0.697 (0.067) | 0.719 (0.064) | 0.693 (0.095) | 0.363 (0.065) | 0.477 (0.048) |
| positives | 0.688 (0.05) | 0.713 (0.049) | 0.683 (0.071) | 0.35 (0.055) | 0.465 (0.04) |
| minority | 0.685 (0.05) | 0.722 (0.043) | 0.678 (0.069) | 0.349 (0.055) | 0.467 (0.042) |
| majority | 0.692 (0.06) | 0.722 (0.045) | 0.686 (0.082) | 0.356 (0.058) | 0.473 (0.046) |
| mpd | 0.703 (0.063) | 0.701 (0.066) | 0.705 (0.091) | 0.366 (0.063) | 0.475 (0.045) |

Supplementary table 4 Mean of accuracy, sensitivity, specificity, precision, and F1 score of all the models tested with the H02 test dataset over 5 folds with standard deviation at the optimum threshold selected at maximum Youden index

| Model | Accuracy | Sensitivity | Specificity | Precision | F1 |
| --- | --- | --- | --- | --- | --- |
| H01 | 0.649 (0.023) | 0.754 (0.035) | 0.618 (0.038) | 0.37 (0.021) | 0.495 (0.015) |
| H02 | 0.701 (0.035) | 0.724 (0.068) | 0.693 (0.061) | 0.415 (0.027) | 0.526 (0.018) |
| H03 | 0.672 (0.059) | 0.682 (0.094) | 0.667 (0.102) | 0.387 (0.038) | 0.489 (0.02) |
| H04 | 0.601 (0.018) | 0.753 (0.038) | 0.556 (0.028) | 0.335 (0.021) | 0.463 (0.023) |
| H05 | 0.647 (0.053) | 0.715 (0.04) | 0.627 (0.081) | 0.367 (0.039) | 0.483 (0.027) |
| H06 | 0.617 (0.057) | 0.714 (0.074) | 0.586 (0.099) | 0.342 (0.015) | 0.461 (0.011) |
| H07 | 0.66 (0.05) | 0.667 (0.124) | 0.656 (0.101) | 0.371 (0.024) | 0.472 (0.021) |
| H08 | 0.647 (0.039) | 0.707 (0.055) | 0.627 (0.065) | 0.362 (0.021) | 0.478 (0.022) |
| H09 | 0.657 (0.044) | 0.649 (0.107) | 0.66 (0.083) | 0.365 (0.029) | 0.463 (0.01) |
| H10 | 0.647 (0.044) | 0.724 (0.079) | 0.623 (0.083) | 0.366 (0.018) | 0.484 (0.015) |
| H11 | 0.614 (0.098) | 0.623 (0.187) | 0.614 (0.182) | 0.339 (0.054) | 0.421 (0.028) |
| GM | 0.676 (0.041) | 0.76 (0.072) | 0.65 (0.074) | 0.396 (0.032) | 0.518 (0.018) |
| unweighted | 0.693 (0.047) | 0.706 (0.066) | 0.689 (0.08) | 0.408 (0.039) | 0.514 (0.017) |
| samples | 0.699 (0.045) | 0.712 (0.071) | 0.695 (0.078) | 0.415 (0.038) | 0.521 (0.011) |
| positives | 0.685 (0.029) | 0.706 (0.05) | 0.678 (0.048) | 0.396 (0.015) | 0.506 (0.005) |
| minority | 0.69 (0.034) | 0.709 (0.052) | 0.685 (0.058) | 0.403 (0.025) | 0.512 (0.007) |
| majority | 0.693 (0.049) | 0.715 (0.074) | 0.687 (0.083) | 0.411 (0.041) | 0.517 (0.013) |
| mpd | 0.699 (0.038) | 0.71 (0.063) | 0.695 (0.067) | 0.413 (0.032) | 0.519 (0.008) |

Supplementary table 5 Mean of accuracy, sensitivity, specificity, precision, and F1 score of all the models tested with the H03 test dataset over 5 folds with standard deviation at the optimum threshold selected at maximum Youden index

| Model | Accuracy | Sensitivity | Specificity | Precision | F1 |
| --- | --- | --- | --- | --- | --- |
| H01 | 0.649 (0.037) | 0.759 (0.085) | 0.622 (0.062) | 0.341 (0.032) | 0.468 (0.022) |
| H02 | 0.697 (0.019) | 0.746 (0.073) | 0.685 (0.023) | 0.376 (0.036) | 0.499 (0.043) |
| H03 | 0.681 (0.071) | 0.713 (0.164) | 0.675 (0.123) | 0.37 (0.059) | 0.475 (0.034) |
| H04 | 0.6 (0.05) | 0.781 (0.075) | 0.552 (0.078) | 0.31 (0.015) | 0.443 (0.023) |
| H05 | 0.588 (0.049) | 0.782 (0.09) | 0.539 (0.08) | 0.304 (0.028) | 0.435 (0.023) |
| H06 | 0.675 (0.047) | 0.688 (0.056) | 0.671 (0.075) | 0.351 (0.029) | 0.464 (0.022) |
| H07 | 0.617 (0.075) | 0.808 (0.069) | 0.569 (0.104) | 0.33 (0.047) | 0.464 (0.041) |
| H08 | 0.641 (0.04) | 0.785 (0.086) | 0.605 (0.057) | 0.338 (0.038) | 0.47 (0.038) |
| H09 | 0.627 (0.056) | 0.745 (0.117) | 0.597 (0.094) | 0.324 (0.028) | 0.447 (0.024) |
| H10 | 0.615 (0.071) | 0.817 (0.081) | 0.565 (0.102) | 0.328 (0.039) | 0.465 (0.032) |
| H11 | 0.532 (0.025) | 0.812 (0.071) | 0.46 (0.048) | 0.277 (0.011) | 0.413 (0.021) |
| GM | 0.672 (0.071) | 0.79 (0.123) | 0.643 (0.118) | 0.375 (0.082) | 0.498 (0.044) |
| unweighted | 0.655 (0.032) | 0.786 (0.078) | 0.623 (0.054) | 0.348 (0.031) | 0.481 (0.028) |
| samples | 0.642 (0.048) | 0.805 (0.103) | 0.602 (0.081) | 0.343 (0.035) | 0.478 (0.026) |
| positives | 0.712 (0.035) | 0.691 (0.061) | 0.717 (0.054) | 0.386 (0.029) | 0.494 (0.024) |
| minority | 0.718 (0.018) | 0.684 (0.066) | 0.727 (0.036) | 0.391 (0.018) | 0.496 (0.019) |
| minority | 0.718 (0.018) | 0.684 (0.066) | 0.727 (0.036) | 0.391 (0.018) | 0.496 (0.019) |
| mpd | 0.694 (0.049) | 0.724 (0.12) | 0.686 (0.088) | 0.375 (0.024) | 0.49 (0.015) |

Supplementary table 6 Mean of accuracy, sensitivity, specificity, precision, and F1 score of all the models tested with the H04 test dataset over 5 folds with standard deviation at the optimum threshold selected at maximum Youden index

| Model | Accuracy | Sensitivity | Specificity | Precision | F1 |
| --- | --- | --- | --- | --- | --- |
| H01 | 0.743 (0.043) | 0.841 (0.069) | 0.725 (0.061) | 0.338 (0.024) | 0.481 (0.022) |
| H02 | 0.712 (0.05) | 0.877 (0.033) | 0.685 (0.059) | 0.317 (0.048) | 0.464 (0.052) |
| H03 | 0.717 (0.035) | 0.842 (0.028) | 0.696 (0.044) | 0.313 (0.031) | 0.456 (0.033) |
| H04 | 0.726 (0.052) | 0.861 (0.051) | 0.704 (0.067) | 0.328 (0.055) | 0.472 (0.051) |
| H05 | 0.66 (0.036) | 0.891 (0.045) | 0.623 (0.043) | 0.28 (0.035) | 0.425 (0.038) |
| H06 | 0.695 (0.033) | 0.842 (0.073) | 0.672 (0.048) | 0.297 (0.031) | 0.437 (0.029) |
| H07 | 0.714 (0.064) | 0.808 (0.024) | 0.699 (0.075) | 0.313 (0.061) | 0.448 (0.063) |
| H08 | 0.674 (0.038) | 0.904 (0.059) | 0.635 (0.054) | 0.29 (0.018) | 0.438 (0.02) |
| H09 | 0.719 (0.058) | 0.821 (0.09) | 0.703 (0.077) | 0.318 (0.058) | 0.454 (0.056) |
| H10 | 0.684 (0.022) | 0.869 (0.017) | 0.653 (0.026) | 0.291 (0.022) | 0.436 (0.026) |
| H11 | 0.692 (0.067) | 0.744 (0.112) | 0.684 (0.093) | 0.288 (0.057) | 0.408 (0.042) |
| GM | 0.715 (0.016) | 0.905 (0.046) | 0.684 (0.027) | 0.319 (0.016) | 0.471 (0.02) |
| unweighted | 0.748 (0.025) | 0.882 (0.041) | 0.726 (0.033) | 0.346 (0.033) | 0.496 (0.034) |
| samples | 0.769 (0.024) | 0.859 (0.029) | 0.754 (0.032) | 0.365 (0.035) | 0.511 (0.033) |
| positives | 0.768 (0.019) | 0.833 (0.031) | 0.757 (0.024) | 0.36 (0.032) | 0.502 (0.032) |
| minority | 0.775 (0.028) | 0.845 (0.042) | 0.763 (0.038) | 0.37 (0.034) | 0.514 (0.032) |
| majority | 0.767 (0.009) | 0.861 (0.032) | 0.751 (0.015) | 0.361 (0.018) | 0.509 (0.023) |
| mpd | 0.777 (0.023) | 0.853 (0.025) | 0.764 (0.029) | 0.373 (0.036) | 0.518 (0.034) |

Supplementary table 7 Mean of accuracy, sensitivity, specificity, precision, and F1 score of all the models tested with the H05 test dataset over 5 folds with standard deviation at the optimum threshold selected at maximum Youden index

| Model | Accuracy | Sensitivity | Specificity | Precision | F1 |
| --- | --- | --- | --- | --- | --- |
| H01 | 0.618 (0.041) | 0.843 (0.055) | 0.57 (0.06) | 0.3 (0.025) | 0.441 (0.022) |
| H02 | 0.658 (0.056) | 0.715 (0.066) | 0.646 (0.081) | 0.309 (0.037) | 0.429 (0.027) |
| H03 | 0.617 (0.118) | 0.711 (0.165) | 0.597 (0.177) | 0.298 (0.075) | 0.402 (0.038) |
| H04 | 0.621 (0.061) | 0.724 (0.089) | 0.6 (0.09) | 0.285 (0.028) | 0.406 (0.021) |
| H05 | 0.656 (0.049) | 0.782 (0.047) | 0.629 (0.068) | 0.317 (0.033) | 0.45 (0.029) |
| H06 | 0.614 (0.064) | 0.76 (0.078) | 0.583 (0.09) | 0.287 (0.033) | 0.414 (0.031) |
| H07 | 0.693 (0.061) | 0.667 (0.108) | 0.699 (0.096) | 0.333 (0.05) | 0.438 (0.029) |
| H08 | 0.663 (0.058) | 0.719 (0.072) | 0.651 (0.082) | 0.314 (0.039) | 0.434 (0.035) |
| H09 | 0.627 (0.117) | 0.736 (0.116) | 0.606 (0.164) | 0.302 (0.06) | 0.42 (0.044) |
| H10 | 0.68 (0.063) | 0.758 (0.094) | 0.664 (0.094) | 0.333 (0.049) | 0.458 (0.032) |
| H11 | 0.618 (0.039) | 0.639 (0.066) | 0.614 (0.059) | 0.265 (0.022) | 0.373 (0.017) |
| GM | 0.692 (0.082) | 0.794 (0.14) | 0.67 (0.129) | 0.361 (0.077) | 0.483 (0.037) |
| unweighted | 0.656 (0.059) | 0.774 (0.082) | 0.632 (0.086) | 0.318 (0.042) | 0.447 (0.034) |
| samples | 0.69 (0.062) | 0.763 (0.085) | 0.675 (0.09) | 0.345 (0.049) | 0.47 (0.033) |
| positives | 0.723 (0.058) | 0.724 (0.089) | 0.722 (0.09) | 0.375 (0.074) | 0.486 (0.034) |
| minority | 0.705 (0.079) | 0.749 (0.116) | 0.696 (0.12) | 0.364 (0.069) | 0.48 (0.039) |
| majority | 0.7 (0.062) | 0.731 (0.088) | 0.694 (0.092) | 0.352 (0.047) | 0.47 (0.029) |
| mpd | 0.676 (0.07) | 0.793 (0.086) | 0.651 (0.101) | 0.34 (0.061) | 0.47 (0.04) |

Supplementary table 8 Mean of accuracy, sensitivity, specificity, precision, and F1 score of all the models tested with the H06 test dataset over 5 folds with standard deviation at the optimum threshold selected at maximum Youden index

| Model | Accuracy | Sensitivity | Specificity | Precision | F1 |
| --- | --- | --- | --- | --- | --- |
| H01 | 0.657 (0.053) | 0.777 (0.131) | 0.621 (0.1) | 0.378 (0.024) | 0.505 (0.025) |
| H02 | 0.666 (0.059) | 0.767 (0.101) | 0.636 (0.103) | 0.386 (0.041) | 0.51 (0.027) |
| H03 | 0.691 (0.054) | 0.693 (0.081) | 0.689 (0.078) | 0.4 (0.052) | 0.505 (0.047) |
| H04 | 0.684 (0.092) | 0.695 (0.119) | 0.678 (0.15) | 0.41 (0.081) | 0.505 (0.033) |
| H05 | 0.655 (0.064) | 0.744 (0.09) | 0.627 (0.105) | 0.376 (0.043) | 0.495 (0.027) |
| H06 | 0.725 (0.055) | 0.717 (0.089) | 0.727 (0.097) | 0.443 (0.041) | 0.543 (0.012) |
| H07 | 0.706 (0.032) | 0.791 (0.102) | 0.682 (0.059) | 0.423 (0.033) | 0.547 (0.021) |
| H08 | 0.66 (0.075) | 0.792 (0.084) | 0.62 (0.122) | 0.389 (0.054) | 0.516 (0.029) |
| H09 | 0.664 (0.044) | 0.725 (0.072) | 0.646 (0.077) | 0.378 (0.032) | 0.494 (0.014) |
| H10 | 0.729 (0.053) | 0.689 (0.102) | 0.739 (0.095) | 0.447 (0.053) | 0.535 (0.009) |
| H11 | 0.672 (0.057) | 0.683 (0.104) | 0.668 (0.096) | 0.383 (0.042) | 0.485 (0.025) |
| GM | 0.727 (0.055) | 0.743 (0.075) | 0.722 (0.087) | 0.448 (0.061) | 0.554 (0.036) |
| unweighted | 0.717 (0.046) | 0.732 (0.058) | 0.712 (0.067) | 0.43 (0.041) | 0.54 (0.035) |
| samples | 0.722 (0.041) | 0.725 (0.074) | 0.721 (0.064) | 0.435 (0.042) | 0.542 (0.035) |
| positives | 0.716 (0.044) | 0.738 (0.03) | 0.709 (0.06) | 0.43 (0.042) | 0.542 (0.032) |
| minority | 0.721 (0.045) | 0.746 (0.054) | 0.713 (0.067) | 0.436 (0.045) | 0.548 (0.034) |
| majority | 0.721 (0.042) | 0.722 (0.077) | 0.721 (0.069) | 0.435 (0.044) | 0.54 (0.032) |
| mpd | 0.728 (0.042) | 0.729 (0.07) | 0.727 (0.063) | 0.442 (0.042) | 0.548 (0.036) |

Supplementary table 9 Mean of accuracy, sensitivity, specificity, precision, and F1 score of all the models tested with the H07 test dataset over 5 folds with standard deviation at the optimum threshold selected at maximum Youden index

| Model | Accuracy | Sensitivity | Specificity | Precision | F1 |
| --- | --- | --- | --- | --- | --- |
| H01 | 0.576 (0.087) | 0.803 (0.115) | 0.481 (0.167) | 0.394 (0.073) | 0.522 (0.063) |
| H02 | 0.654 (0.059) | 0.623 (0.21) | 0.683 (0.161) | 0.46 (0.102) | 0.497 (0.052) |
| H03 | 0.604 (0.093) | 0.776 (0.216) | 0.538 (0.213) | 0.425 (0.094) | 0.525 (0.04) |
| H04 | 0.572 (0.045) | 0.845 (0.099) | 0.454 (0.106) | 0.388 (0.025) | 0.531 (0.037) |
| H05 | 0.639 (0.048) | 0.7 (0.08) | 0.607 (0.093) | 0.423 (0.021) | 0.527 (0.035) |
| H06 | 0.643 (0.059) | 0.693 (0.101) | 0.625 (0.111) | 0.436 (0.076) | 0.527 (0.047) |
| H07 | 0.679 (0.066) | 0.711 (0.083) | 0.661 (0.11) | 0.469 (0.066) | 0.562 (0.052) |
| H08 | 0.656 (0.085) | 0.725 (0.122) | 0.613 (0.188) | 0.448 (0.04) | 0.549 (0.035) |
| H09 | 0.605 (0.053) | 0.722 (0.144) | 0.557 (0.13) | 0.4 (0.043) | 0.506 (0.032) |
| H10 | 0.618 (0.067) | 0.722 (0.135) | 0.578 (0.117) | 0.416 (0.075) | 0.518 (0.067) |
| H11 | 0.556 (0.042) | 0.785 (0.105) | 0.464 (0.08) | 0.373 (0.047) | 0.502 (0.046) |
| GM | 0.616 (0.096) | 0.796 (0.083) | 0.538 (0.17) | 0.423 (0.061) | 0.547 (0.038) |
| unweighted | 0.652 (0.075) | 0.713 (0.099) | 0.623 (0.134) | 0.444 (0.056) | 0.542 (0.04) |
| samples | 0.616 (0.039) | 0.779 (0.14) | 0.549 (0.084) | 0.412 (0.03) | 0.535 (0.041) |
| positives | 0.621 (0.07) | 0.762 (0.192) | 0.566 (0.159) | 0.424 (0.068) | 0.532 (0.058) |
| minority | 0.6 (0.059) | 0.815 (0.152) | 0.511 (0.127) | 0.406 (0.04) | 0.536 (0.043) |
| majority | 0.629 (0.044) | 0.75 (0.109) | 0.581 (0.072) | 0.421 (0.041) | 0.535 (0.041) |
| mpd | 0.609 (0.037) | 0.791 (0.143) | 0.534 (0.084) | 0.407 (0.029) | 0.534 (0.041) |

Supplementary table 10 Mean of accuracy, sensitivity, specificity, precision, and F1 score of all the models tested with the H08 test dataset over 5 folds with standard deviation at the optimum threshold selected at maximum Youden index

| Model | Accuracy | Sensitivity | Specificity | Precision | F1 |
| --- | --- | --- | --- | --- | --- |
| H01 | 0.69 (0.038) | 0.759 (0.037) | 0.655 (0.056) | 0.528 (0.039) | 0.622 (0.034) |
| H02 | 0.662 (0.034) | 0.802 (0.09) | 0.592 (0.089) | 0.502 (0.037) | 0.614 (0.015) |
| H03 | 0.677 (0.028) | 0.778 (0.088) | 0.628 (0.079) | 0.516 (0.038) | 0.617 (0.017) |
| H04 | 0.656 (0.025) | 0.81 (0.057) | 0.579 (0.047) | 0.493 (0.028) | 0.612 (0.022) |
| H05 | 0.637 (0.046) | 0.827 (0.088) | 0.54 (0.115) | 0.481 (0.035) | 0.605 (0.014) |
| H06 | 0.669 (0.025) | 0.771 (0.09) | 0.619 (0.071) | 0.507 (0.036) | 0.609 (0.022) |
| H07 | 0.667 (0.054) | 0.795 (0.047) | 0.601 (0.105) | 0.508 (0.043) | 0.618 (0.024) |
| H08 | 0.713 (0.032) | 0.807 (0.055) | 0.666 (0.048) | 0.551 (0.039) | 0.653 (0.031) |
| H09 | 0.679 (0.017) | 0.722 (0.065) | 0.658 (0.051) | 0.517 (0.029) | 0.601 (0.017) |
| H10 | 0.678 (0.046) | 0.785 (0.11) | 0.626 (0.104) | 0.521 (0.065) | 0.62 (0.036) |
| H11 | 0.644 (0.042) | 0.627 (0.113) | 0.655 (0.106) | 0.484 (0.051) | 0.539 (0.037) |
| GM | 0.696 (0.045) | 0.838 (0.031) | 0.624 (0.071) | 0.532 (0.042) | 0.65 (0.032) |
| unweighted | 0.693 (0.019) | 0.815 (0.074) | 0.633 (0.039) | 0.528 (0.026) | 0.639 (0.029) |
| samples | 0.706 (0.031) | 0.772 (0.054) | 0.674 (0.055) | 0.547 (0.045) | 0.638 (0.029) |
| positives | 0.697 (0.026) | 0.791 (0.062) | 0.651 (0.021) | 0.533 (0.031) | 0.636 (0.037) |
| minority | 0.704 (0.029) | 0.791 (0.062) | 0.662 (0.047) | 0.542 (0.042) | 0.642 (0.034) |
| majority | 0.71 (0.021) | 0.76 (0.081) | 0.685 (0.051) | 0.551 (0.033) | 0.636 (0.03) |
| mpd | 0.704 (0.02) | 0.787 (0.063) | 0.662 (0.015) | 0.539 (0.024) | 0.64 (0.034) |

Supplementary table 11 Mean of accuracy, sensitivity, specificity, precision, and F1 score of all the models tested with the H09 test dataset over 5 folds with standard deviation at the optimum threshold selected at maximum Youden index

| Model | Accuracy | Sensitivity | Specificity | Precision | F1 |
| --- | --- | --- | --- | --- | --- |
| H01 | 0.639 (0.044) | 0.863 (0.035) | 0.584 (0.057) | 0.344 (0.041) | 0.49 (0.043) |
| H02 | 0.707 (0.024) | 0.764 (0.083) | 0.693 (0.05) | 0.385 (0.039) | 0.51 (0.034) |
| H03 | 0.663 (0.044) | 0.831 (0.075) | 0.621 (0.074) | 0.358 (0.048) | 0.496 (0.034) |
| H04 | 0.68 (0.009) | 0.746 (0.031) | 0.664 (0.009) | 0.357 (0.025) | 0.482 (0.025) |
| H05 | 0.658 (0.023) | 0.842 (0.029) | 0.612 (0.031) | 0.352 (0.036) | 0.496 (0.038) |
| H06 | 0.692 (0.042) | 0.748 (0.069) | 0.676 (0.066) | 0.368 (0.018) | 0.493 (0.027) |
| H07 | 0.667 (0.046) | 0.76 (0.065) | 0.645 (0.067) | 0.353 (0.053) | 0.478 (0.05) |
| H08 | 0.637 (0.045) | 0.863 (0.027) | 0.581 (0.058) | 0.342 (0.043) | 0.488 (0.045) |
| H09 | 0.719 (0.028) | 0.76 (0.049) | 0.708 (0.039) | 0.395 (0.02) | 0.519 (0.02) |
| H10 | 0.714 (0.042) | 0.748 (0.052) | 0.706 (0.061) | 0.393 (0.055) | 0.512 (0.043) |
| H11 | 0.627 (0.052) | 0.758 (0.081) | 0.591 (0.087) | 0.32 (0.008) | 0.449 (0.018) |
| GM | 0.67 (0.067) | 0.871 (0.058) | 0.62 (0.098) | 0.371 (0.058) | 0.517 (0.046) |
| unweighted | 0.681 (0.052) | 0.817 (0.109) | 0.649 (0.089) | 0.373 (0.053) | 0.506 (0.037) |
| samples | 0.693 (0.044) | 0.805 (0.084) | 0.666 (0.074) | 0.381 (0.051) | 0.512 (0.036) |
| positives | 0.711 (0.046) | 0.766 (0.074) | 0.699 (0.075) | 0.395 (0.061) | 0.516 (0.04) |
| minority | 0.697 (0.043) | 0.805 (0.093) | 0.671 (0.077) | 0.385 (0.054) | 0.515 (0.034) |
| majority | 0.696 (0.049) | 0.8 (0.1) | 0.672 (0.085) | 0.385 (0.057) | 0.514 (0.037) |
| mpd | 0.694 (0.039) | 0.812 (0.083) | 0.666 (0.069) | 0.382 (0.051) | 0.515 (0.035) |

Supplementary table 12 Mean of accuracy, sensitivity, specificity, precision, and F1 score of all the models tested with the H10 test dataset over 5 folds with standard deviation at the optimum threshold selected at maximum Youden index

| Model | Accuracy | Sensitivity | Specificity | Precision | F1 |
| --- | --- | --- | --- | --- | --- |
| H01 | 0.779 (0.021) | 0.832 (0.025) | 0.686 (0.071) | 0.823 (0.028) | 0.827 (0.013) |
| H02 | 0.706 (0.016) | 0.758 (0.039) | 0.614 (0.059) | 0.773 (0.023) | 0.765 (0.02) |
| H03 | 0.671 (0.018) | 0.66 (0.034) | 0.691 (0.035) | 0.787 (0.016) | 0.717 (0.02) |
| H04 | 0.718 (0.017) | 0.736 (0.064) | 0.688 (0.075) | 0.806 (0.03) | 0.767 (0.026) |
| H05 | 0.734 (0.02) | 0.806 (0.044) | 0.608 (0.067) | 0.782 (0.02) | 0.793 (0.02) |
| H06 | 0.715 (0.041) | 0.798 (0.078) | 0.569 (0.04) | 0.762 (0.015) | 0.779 (0.044) |
| H07 | 0.609 (0.082) | 0.583 (0.157) | 0.656 (0.063) | 0.741 (0.031) | 0.644 (0.113) |
| H08 | 0.698 (0.019) | 0.734 (0.063) | 0.632 (0.097) | 0.779 (0.028) | 0.754 (0.027) |
| H09 | 0.713 (0.029) | 0.841 (0.073) | 0.491 (0.047) | 0.741 (0.008) | 0.787 (0.031) |
| H10 | 0.851 (0.009) | 0.861 (0.016) | 0.834 (0.026) | 0.9 (0.012) | 0.88 (0.009) |
| H11 | 0.61 (0.055) | 0.641 (0.213) | 0.553 (0.232) | 0.728 (0.049) | 0.659 (0.114) |
| GM | 0.843 (0.014) | 0.839 (0.036) | 0.849 (0.029) | 0.907 (0.01) | 0.871 (0.015) |
| unweighted | 0.765 (0.021) | 0.797 (0.067) | 0.711 (0.072) | 0.828 (0.026) | 0.81 (0.025) |
| samples | 0.811 (0.013) | 0.807 (0.02) | 0.819 (0.035) | 0.886 (0.016) | 0.844 (0.011) |
| positives | 0.838 (0.014) | 0.816 (0.025) | 0.877 (0.008) | 0.92 (0.004) | 0.865 (0.013) |
| minority | 0.833 (0.022) | 0.838 (0.047) | 0.826 (0.03) | 0.893 (0.014) | 0.864 (0.021) |
| majority | 0.803 (0.015) | 0.803 (0.031) | 0.801 (0.036) | 0.875 (0.013) | 0.838 (0.015) |
| mpd | 0.822 (0.02) | 0.819 (0.037) | 0.827 (0.03) | 0.892 (0.011) | 0.853 (0.019) |

Supplementary table 13 Mean of accuracy, sensitivity, specificity, precision, and F1 score of all the models tested with the H10 test dataset over 5 folds with standard deviation at the optimum threshold selected at maximum Youden index

| Model | Accuracy | Sensitivity | Specificity | Precision | F1 |
| --- | --- | --- | --- | --- | --- |
| H01 | 0.626 (0.136) | 0.827 (0.165) | 0.596 (0.152) | 0.216 (0.094) | 0.334 (0.129) |
| H02 | 0.684 (0.126) | 0.783 (0.098) | 0.68 (0.142) | 0.266 (0.158) | 0.369 (0.175) |
| H03 | 0.691 (0.065) | 0.815 (0.101) | 0.671 (0.086) | 0.242 (0.104) | 0.365 (0.13) |
| H04 | 0.704 (0.101) | 0.708 (0.16) | 0.711 (0.128) | 0.26 (0.139) | 0.35 (0.144) |
| H05 | 0.714 (0.073) | 0.681 (0.143) | 0.719 (0.097) | 0.255 (0.149) | 0.347 (0.152) |
| H06 | 0.739 (0.091) | 0.683 (0.175) | 0.735 (0.113) | 0.251 (0.087) | 0.364 (0.112) |
| H07 | 0.7 (0.101) | 0.734 (0.089) | 0.689 (0.129) | 0.243 (0.107) | 0.356 (0.126) |
| H08 | 0.618 (0.104) | 0.87 (0.071) | 0.591 (0.106) | 0.224 (0.117) | 0.341 (0.15) |
| H09 | 0.615 (0.215) | 0.778 (0.166) | 0.605 (0.248) | 0.24 (0.155) | 0.333 (0.161) |
| H10 | 0.689 (0.102) | 0.806 (0.154) | 0.667 (0.126) | 0.243 (0.099) | 0.365 (0.125) |
| H11 | 0.585 (0.134) | 0.841 (0.11) | 0.561 (0.154) | 0.211 (0.118) | 0.318 (0.138) |
| GM | 0.786 (0.068) | 0.728 (0.179) | 0.785 (0.088) | 0.303 (0.089) | 0.423 (0.11) |
| unweighted | 0.714 (0.111) | 0.739 (0.131) | 0.715 (0.125) | 0.268 (0.152) | 0.372 (0.17) |
| samples | 0.724 (0.082) | 0.728 (0.117) | 0.719 (0.099) | 0.257 (0.129) | 0.37 (0.149) |
| positives | 0.78 (0.046) | 0.697 (0.131) | 0.784 (0.061) | 0.289 (0.122) | 0.402 (0.137) |
| minority | 0.761 (0.07) | 0.722 (0.154) | 0.757 (0.094) | 0.276 (0.122) | 0.394 (0.142) |
| majority | 0.722 (0.07) | 0.727 (0.114) | 0.716 (0.079) | 0.251 (0.12) | 0.366 (0.146) |
| mpd | 0.746 (0.08) | 0.716 (0.108) | 0.745 (0.095) | 0.276 (0.14) | 0.386 (0.159) |

# Box Plots


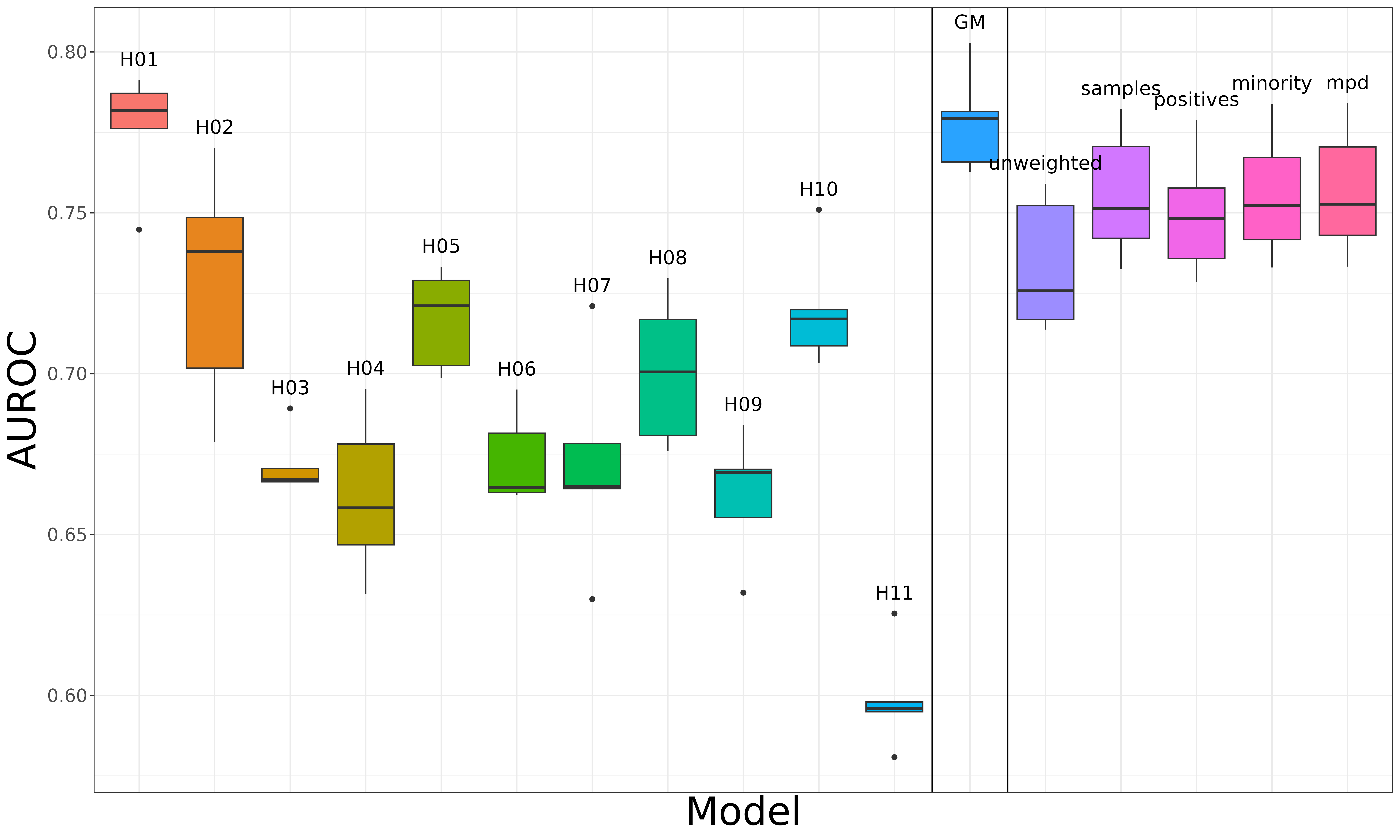
 **Fig S1** Models were tested using test data from Hospital H01


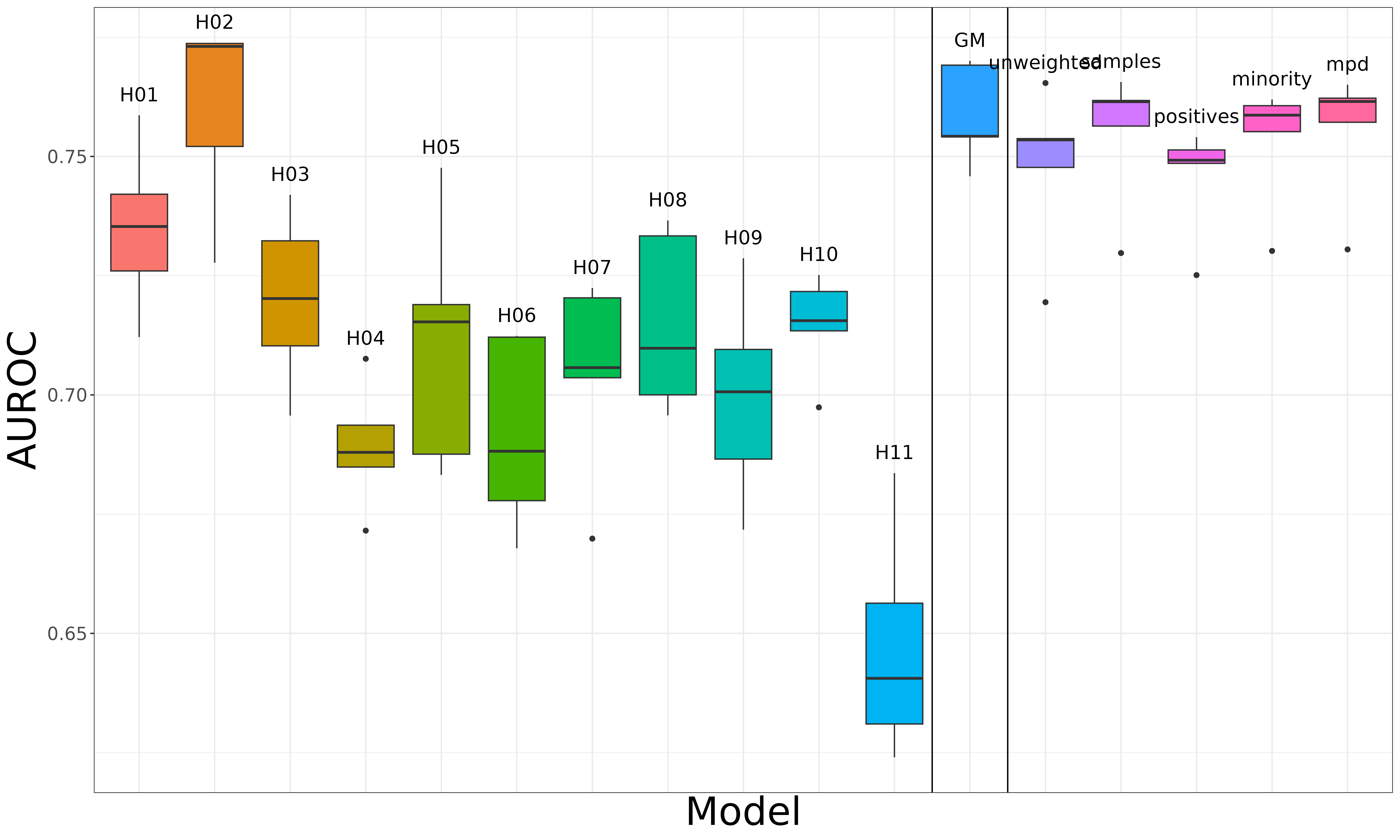


**Fig S2** Models were tested using test data from Hospital H02


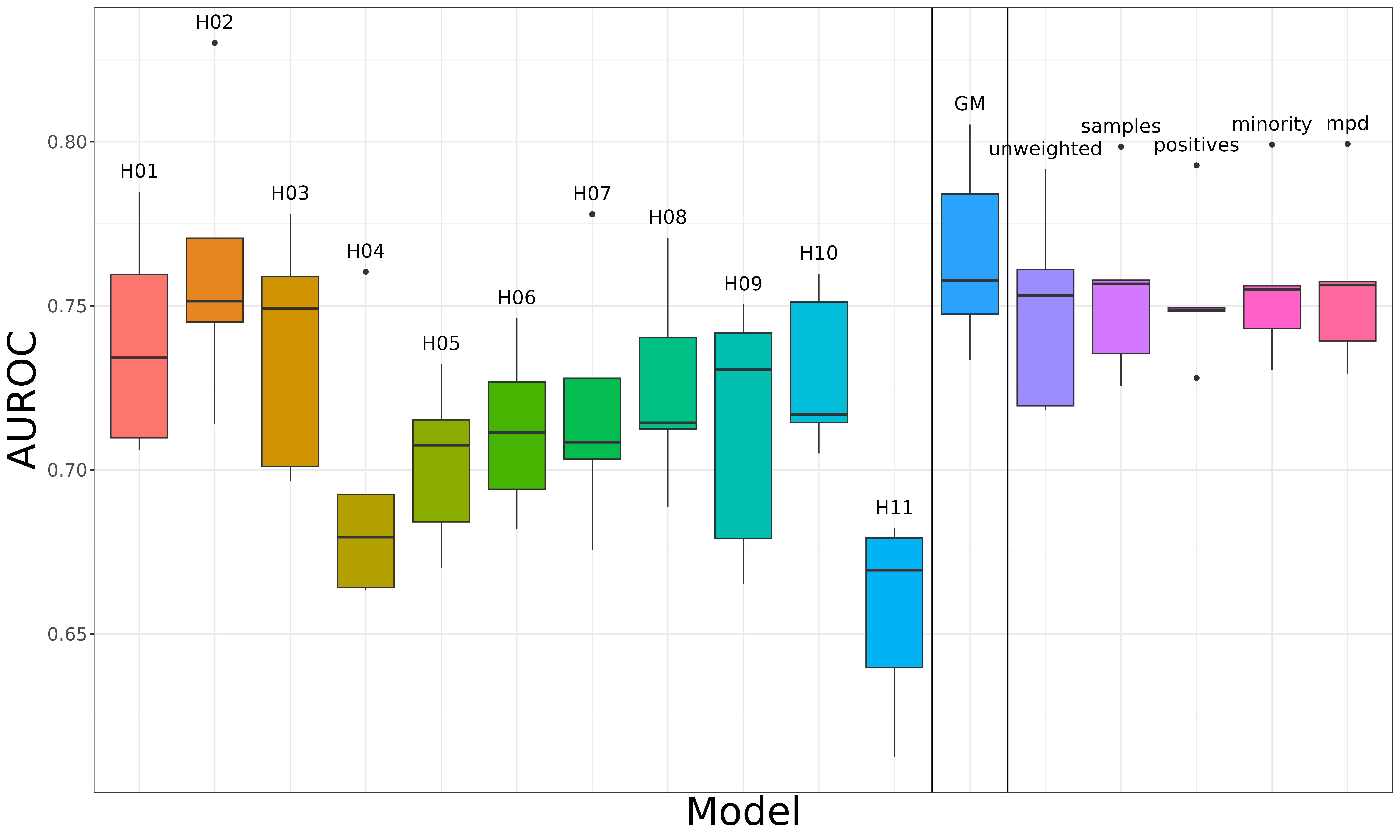
**Fig S3** Models were tested using test data from Hospital H03


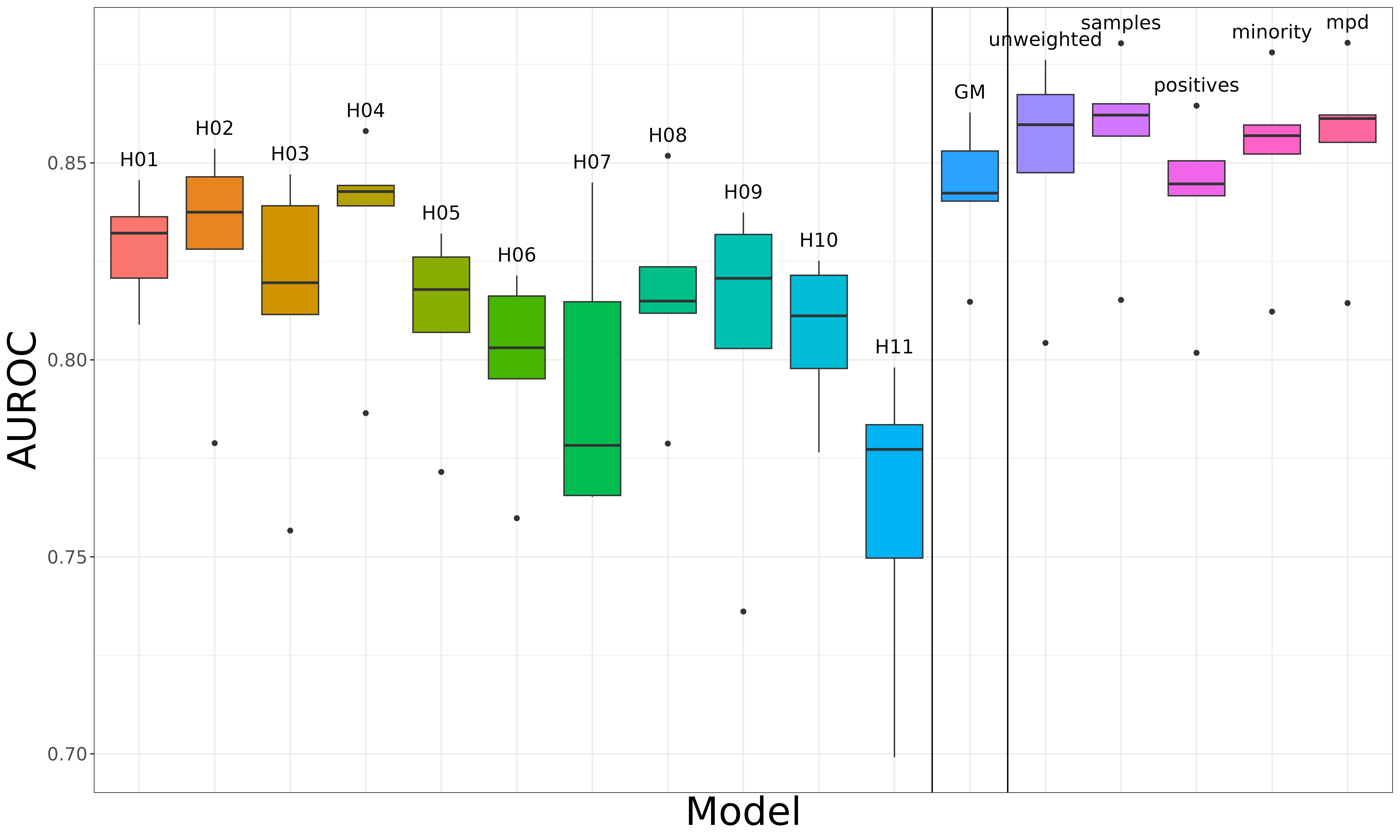
**Fig S4** Models were tested using test data from Hospital H04


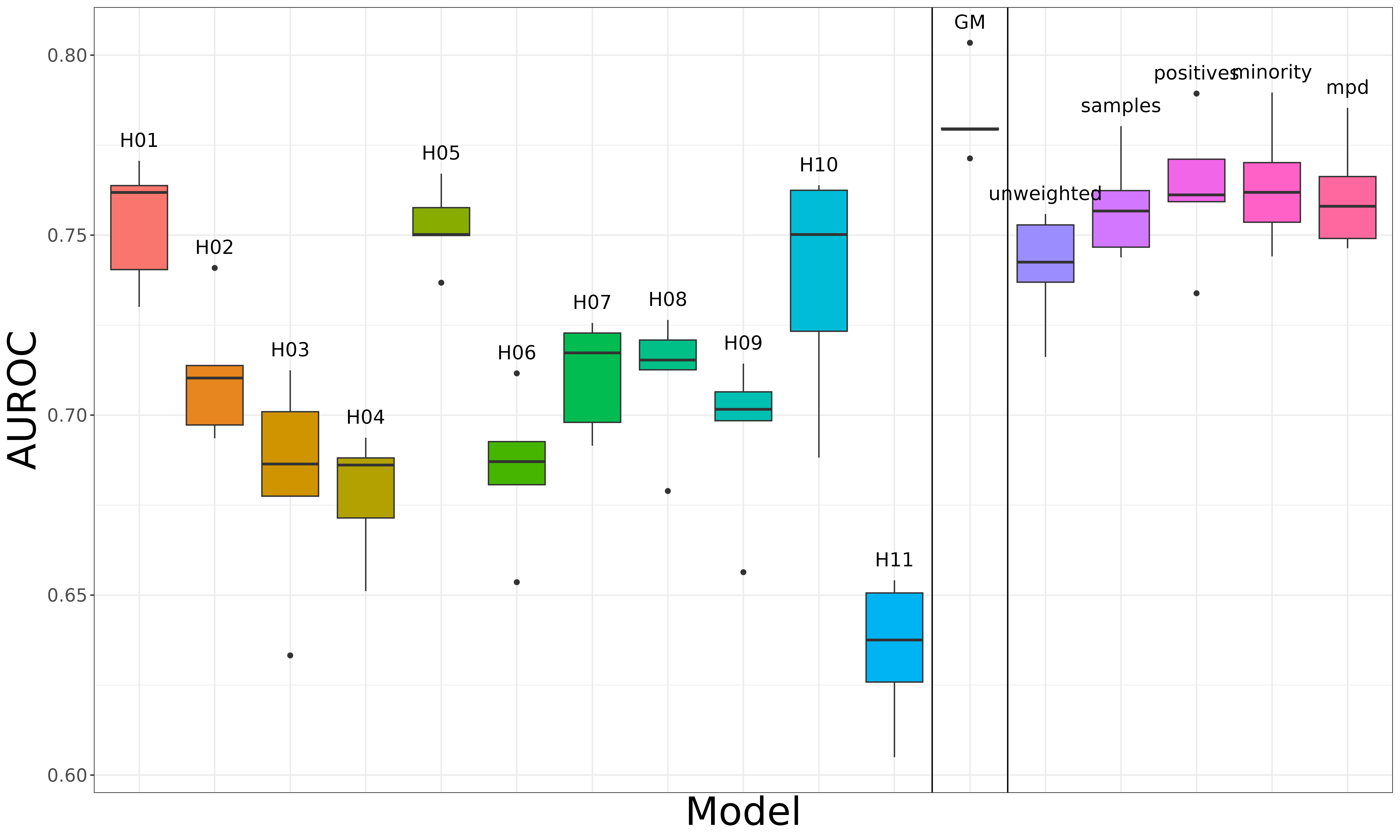


**Fig S5** Models were tested using test data from Hospital H05


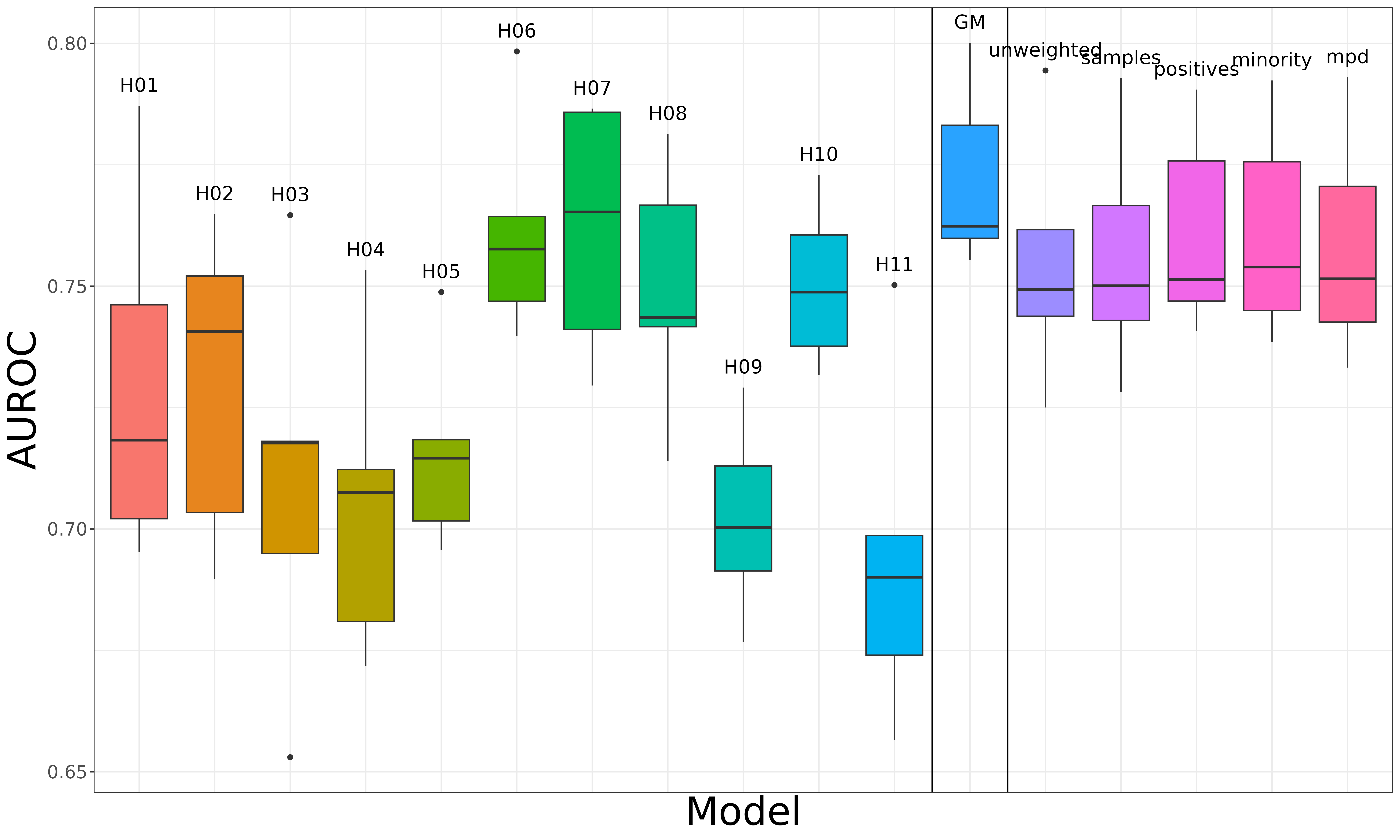


**Fig S6** Models were tested using test data from Hospital H06


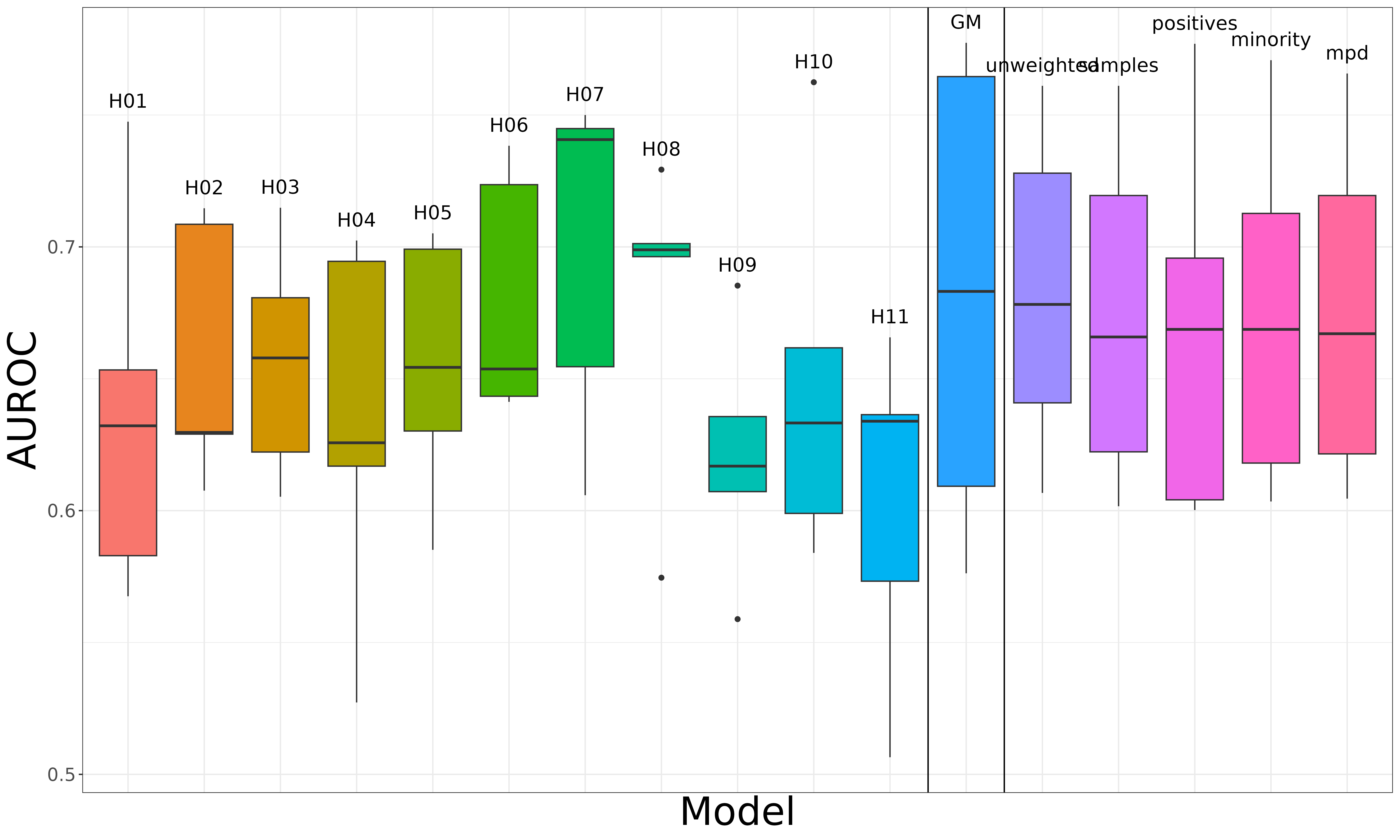


**Fig S7** Models were tested using test data from Hospital H07


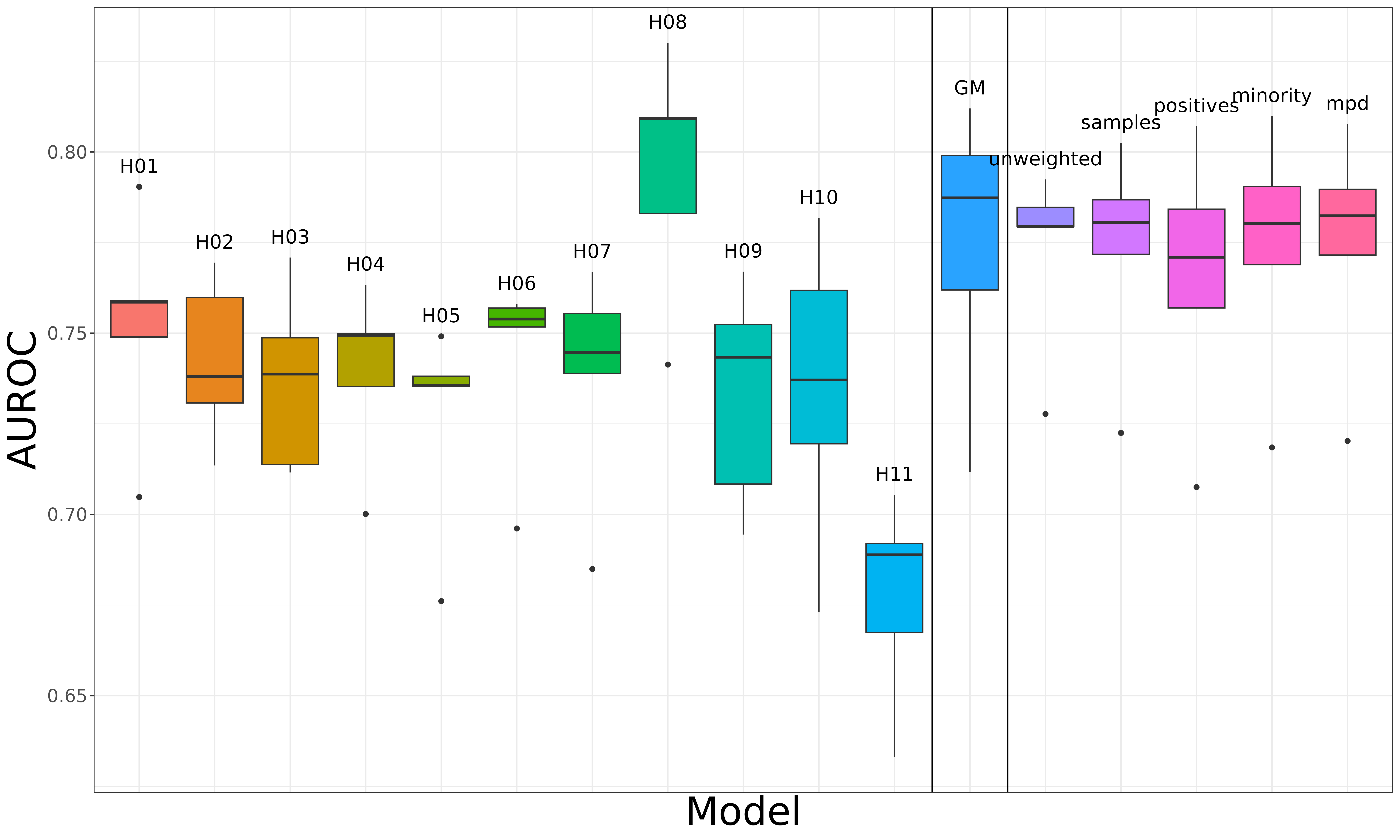


**Fig S8** Models were tested using test data from Hospital H08


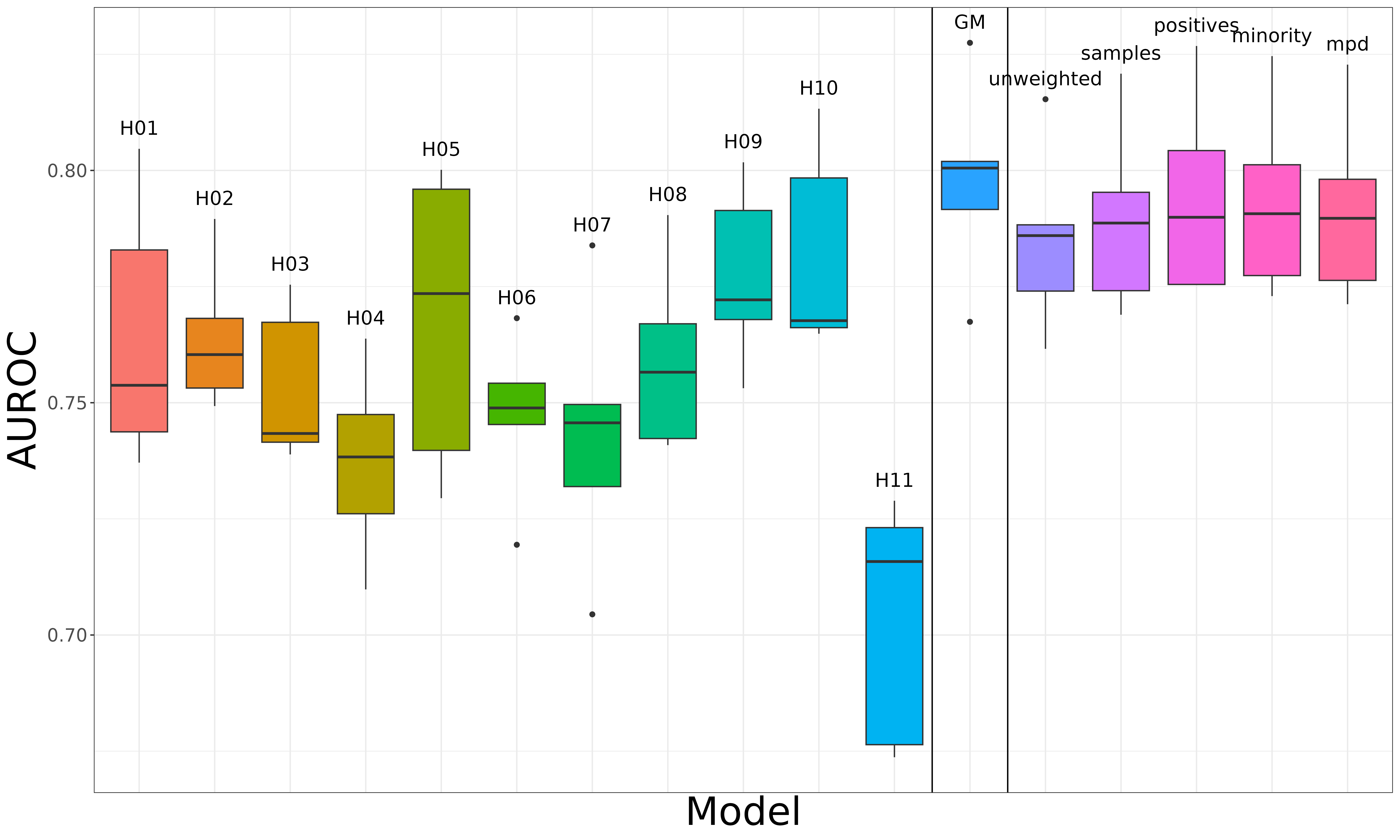


**Fig S9** Models were tested using test data from Hospital H09


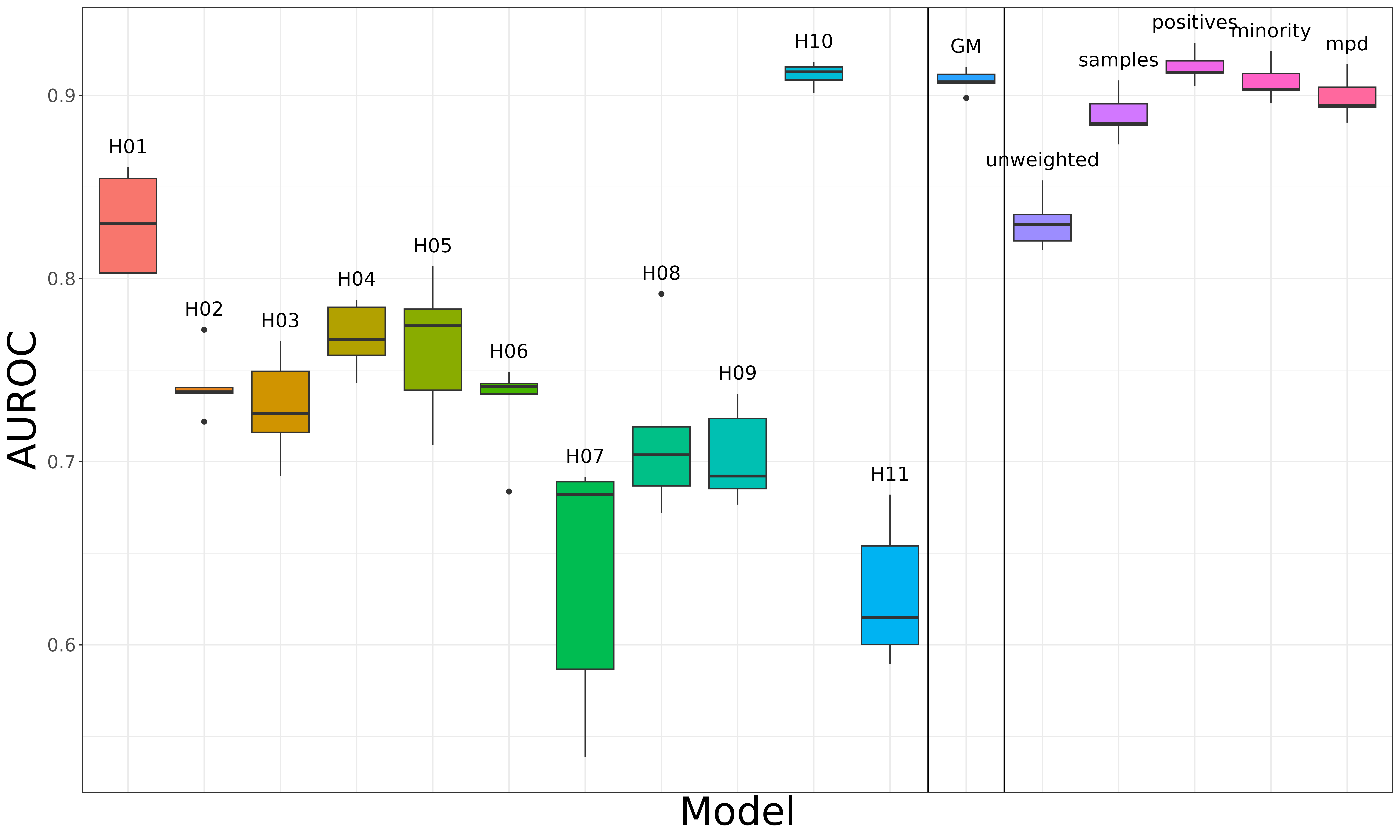


**Fig S10** Models were tested using test data from Hospital H10


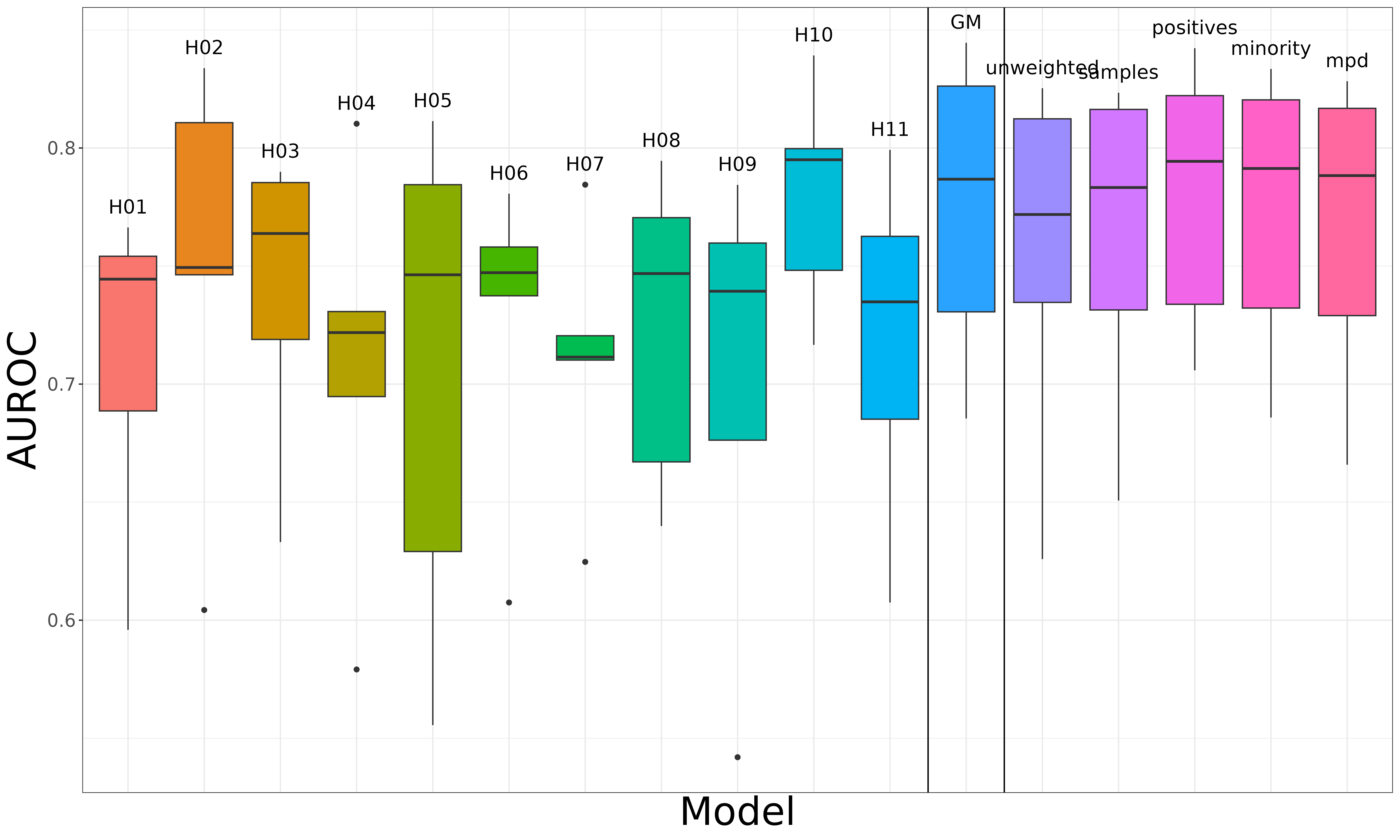


**Fig S11** Models were tested using test data from Hospital H11





**Fig S12** A heat map representaion of all models perfornmace in terms of mean AUROC [95% confidence interval] while tested with all datasets
